# *08SG2*/*OsBAK1* regulates grain size and number, and functions differently in *Indica* and *Japonica* backgrounds in rice

**DOI:** 10.1186/s12284-017-0165-2

**Published:** 2017-05-25

**Authors:** Hua Yuan, Shijun Fan, Juan Huang, Shijie Zhan, Shifu Wang, Peng Gao, Weilan Chen, Bin Tu, Bingtian Ma, Yuping Wang, Peng Qin, Shigui Li

**Affiliations:** 1 Rice Research Institute of Sichuan Agricultural University, Chengdu Wenjiang, Sichuan 611130 China; 2 State Key Laboratory of Hybrid Rice, Sichuan Agricultural University, Chengdu Wenjiang, Sichuan 611130 China; 3 Institute of Characteristic Crops Research, Chongqing Academy of Agricultural Sciences, Yongchuan, Chongqing 402160 China

**Keywords:** Rice, *08SG2/OsBAK1*, Grain size, Grain number, BR, Cell proliferation

## Abstract

**Background:**

Both grain size and grain number are significant for rice yield. In the past decade, a number of genes related to grain size and grain number have been documented, however, the regulatory mechanisms underlying them remains ambiguous.

**Results:**

We identified a rice *small grain* (*sg2*) mutant in an EMS mutant library generated from an *indica* variety, *Shuhui498*. Using the MutMap gene mapping strategy, we identified two linkage regions on chromosome 7 and 8, respectively, consistent with the segregation ratios in the F_2_ population. We focused on the linkage region on chromosome 8, and named this locus as *08sg2*. One of three SNPs identified in the linkage region was located in an exon of *OsBAK1*, leading to a nonsynonymous mutation in the kinase domain. The plant harboring the mutant version *08sg2* locus exhibited a decreased grain size, grain number and plant height. Cytological analysis indicated that *08SG2* regulated spikelet hull development by affecting cell proliferation. The grain size and number of knockout mutants of *OsBAK1* in the *japonica* background were significantly decreased, but less so than in *08sg2*, supporting the idea that the SNP in *OsBAK1* was responsible for the *08sg2* phenotype, but that *08SG2/OsBAK*1 function differently in *indica* and *japonica* backgrounds. *08sg2* was insensitive to 24-epiBL, and the expression of BR-related genes was obviously altered in *08sg2*. The proportionally decreased grain length when *08sg2* and *GS3* were combined indicate that *08SG2* and *GS3* regulate grain length independently.

**Conclusions:**

Our work shows that *08SG2/OsBAK1* is important for rice yield in both *indica* and *japonica* backgrounds, by regulating grain size and grain number, and the function of *08SG2/OsBAK1* is obviously affected by genetic background. The amino acid substituted in *08sg2* is highly conserved among different species, supporting the idea that it is important for the molecular function of *08SG2/OsBAK1*. Together, our work is helpful for fully understanding the function of *08SG2/OsBAK1*.

**Electronic supplementary material:**

The online version of this article (doi:10.1186/s12284-017-0165-2) contains supplementary material, which is available to authorized users.

## Background

Grain size and grain number are the most important factors determining grain yield in rice. So far, a lot of genes related to grain size and number have been documented. Regarding grain size, multiple signaling pathways that influence grain size have been identified in rice (Li and Li 2014, 2016; Zheng et al. 2015; Zuo and Li 2014), including the ubiquitination-mediated proteasomal degradation pathway, G-protein signaling pathway, the mitogen-activated protein kinase signaling pathway, transcriptional regulatory factors and phytohormone pathways. The ubiquitination-mediated proteasomal degradation pathway includes *GW2* (Song et al. 2007) and *GW5/qSW5* (Shomura et al. 2008; Weng et al. 2008), and the G-protein signaling pathway includes *GS3* and *DEP1. GS3* is a homolog of *AGG3*, the G protein γ-subunit of Arabidopsis (Li et al. 2012), which encodes a putative transmembrane protein and functions as a negative regulator for grain size (Fan et al. 2006; Mao et al. 2010). *DEP1* encodes a plant-specific G protein γ subunit and mutants in it have short grains (Huang et al. 2009). The mitogen-activated protein kinase (MAPK) signaling pathway includes *OsMKK4* and *OsMAPK6. OsMAPK6* interacts strongly with *OsMKK4*, indicating that the *OsMKK4-OsMAPK6* cascade influences grain size in rice (Duan et al. 2014; Liu et al. 2015). The transcriptional regulatory factors pathway includes *GW8*, *GLW7* and *GS2/GL2/GLW2/OsGRF4. GW8* and *GLW7* encode the plant-specific transcription factors *OsSPL16* and *OsSPL13* (Si et al. 2016; Wang et al. 2012), respectively. *GS2/GL2/GLW2* encodes a transcription factor Growth-Regulating Factor 4 (*OsGRF4*), which regulates grain length and width mainly by promoting cell expansion (Che et al. 2015; Duan et al. 2015; Hu et al. 2015; Li et al. 2016). Another important signaling pathway controlling grain size is phytohormone pathway, which has been proven to play a crucial role in rice development, especially in the course of grain growth. For example, *GS5*, a putative serine carboxypeptidase, functions as a positive regulator of grain size (Li et al. 2011), and inhibits the interaction between *OsBAK1-7* and *OsMSBP1*, suggesting that *GS5* might regulate grain size through the brassinosteroids (BR) signaling pathway (Xu et al. 2015). *GL3.1/qGL3* encodes a putative protein phosphatase with a Kelch-like repeat domain (*OsPPKL1*), and may function as a negative regulator through BR signaling in rice (Hu et al. 2012; Qi et al. 2012; Zhang et al. 2012). Regarding grain number, many QTLs and genes have been cloned, such as *Gn1a* (Ashikari et al. 2005), *DEP1* (Yan et al. 2007), *DEP3* (Qiao et al. 2011), *GNP1* (Wu et al. 2016b) and *GAD1* (Jin et al. 2016). However, as complex agronomic traits, the regulatory mechanism of grain size and number remains largely unknown.

*OsBAK1*, as a *BRI1*-associated receptor kinase (*BAK1*), has been proven to participate in BR signal transduction and regulate plant architecture in rice (Li et al. 2009; Shimin et al. 2014), but no mutants have been identified so far, and there was no comprehensive investigation of *OsBAK1* function on yield-related traits. In this study, we identified a mutant of *OsBAK1* in the *indica* background, named *08sg2*, which exhibited a decreased plant height, grain size, grain number and panicle length. Loss of function of *OsBAK1* in the *japonica* background exhibited significantly decreased grain size and number, but less so than in the *indica* background. Our results show that the reduced grain length of *08sg2* was due to decreased cell proliferation. *08sg2* was insensitive to BR, and *08SG2* affected the expression of BR-related genes. Pyramiding analysis indicated that *08SG2/OsBAK1* regulate grain length independently of *GS3*.

## Results

### Isolation the *08sg2* mutant from a *small grain* (*sg2*) mutant

We identified a *small grain* (*sg2*) mutant from an EMS mutant library generated from an *indica* variety *Shuhui498* (R498), which is an excellent restorer of hybrid rice. The *sg2* mutant showed a significant change of plant height, grain size and panicle architecture (Additional file 1: Figure S1, Additional file 1: Table S1). To identify the gene responsible for the *sg2* phenotype, we generated a F_2_ population from a cross between *sg2* mutant and the parental line R498. Only 28 plants, about one-sixteenth of the plants (χ^2^_(15:1)_ = 1.45 < (χ^2^_(0.05,1)_ = 3.84) showed short grain length (Additional file 1: Figure S2A), suggesting that two recessive loci were responsible for the *sg2* phenotype. Subsequently, we applied the MutMap strategy for gene mapping (Abe et al. 2012). Consistent with the segregation ratio, we identified two linkage regions on chromosome 7 and 8, respectively (Additional file 1: Figure S2B). These results suggest that the *sg2* mutant phenotype is due to two recessive mutations, simultaneously. In this study, we focused on the linkage region on chromosome 8, and named this locus *08sg2*.

Three linkage SNPs (SNP index = 1) on chromosome 8 were identified (Additional file 1: Table S2), which were located in *LOC_Os08g05980*, *LOC_Os08g07760* and *LOC_Os08g08000*, respectively. RNA-sequencing showed that only *LOC_Os08g07760* and *LOC_Os08g08000* were expressed in young panicles, furthermore, only the SNP2 in *LOC_Os08g07760* led to a nonsynonymous mutation of a glycine (GGT) to an aspartic acid (GAT) (Additional file 1: Table S2). Therefore, we considered *LOC_Os08g07760* as the best candidate gene for *08SG2*, which was identified as a *BRI1*-associated receptor kinase (*BAK1*) that participates in BR signal transduction (Li et al. 2009). Then, we obtained plants harboring only the *08sg2* locus using markers based on the SNP in *08SG2*/*OsBAK1*, and generated a F_2_ segregation population from a cross between *08sg2* and R498. The SNP in *LOC_Os08g07760* was co-segregated with the plants with extremely short grain from F_2_ population (Additional file 1: Figure S3), confirming *LOC_Os08g07760* as the best candidate gene for *08SG2*.

### The *08sg2* mutant exhibits a decreased grain size and grain number

**Fig. 1:**
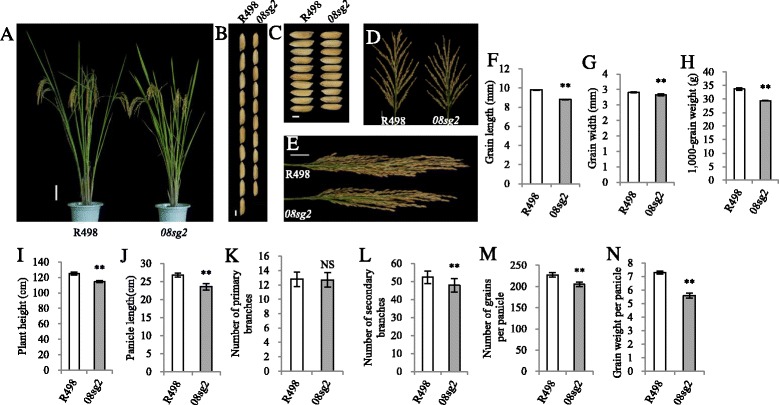
Comparisons of phenotype between R498 and *08sg2*. **a** The phenotype of R498 and *08sg2* at reproductive stage. Scale bar, 10 cm. **b**, **c** Comparisons of the grain length and grain width between R498 and *08sg2*. Scale bar, 3 mm. **d**, **e** The panicle morphology of R498 and *08sg2*. Scale bar, 3 cm. **f** Grain length. **g** Grain width. **h** 1,000-grain weight. **i** Plant height. **j** Panicle length. **k** Number of primary branches. **l** Number of secondary branches. **m** Number of grains per panicle. **n** Grain weight per panicle. All phenotypic data in F–N were measured from plants grown in paddies under normal cultivation conditions with three-time repeats. Data are given as means ± SD. Student’s t-test was used to generate the *P* values; ** and NS indicate *P* < 0.01 and no significant differences, respectively

To eliminate other potential mutations, *08sg2* was backcrossed with R498 three times. Compared with R498, the plant height of *08sg2* was reduced (Fig. 1a, i), which was mainly due to the shortened panicle length and uppermost internode (Additional file 1: Figure S4). The grain length and width of *08sg2* was reduced, 10.2% and 2.7%, respectively, as a result, the 1000-grain weight of *08sg2* was reduced 12.8% (Fig. 1b, c, f, g, h). We dynamically investigated the fresh and dry weight of endosperm between *08sg2* and R498. Both fresh and dry weight of *08sg2* endosperm was significantly less than those of R498 from 9 days after fertilization (Additional file 1: Figure S5), indicating that *08sg2* had a slower grain filling rate. In addition, the panicle length of *08sg2* was reduced 11.9% (Fig. 1e, j). The number of primary branches had no significant difference from that of R498 (Fig. 1d, k), but the number of secondary branches was reduced 8.6%, resulting in a 9.7% reduction in grain number per panicle (Fig. 1l, m). Consistent with this, the expression of *OsCLV1* and *OsCLV2*, which are negative regulators of panicle branching (Chu et al. 2006; Suzaki et al. 2004), were significantly up-regulated in *08sg2* (Additional file 1: Figure S6). Due to the reduction of both grain size and grain number, the grain weight per panicle of *08sg2* was significantly reduced, up to 23.3% (Fig. 1n).

### *08SG2* regulates the development of spikelet hull by affecting cell proliferation

**Fig. 2:**
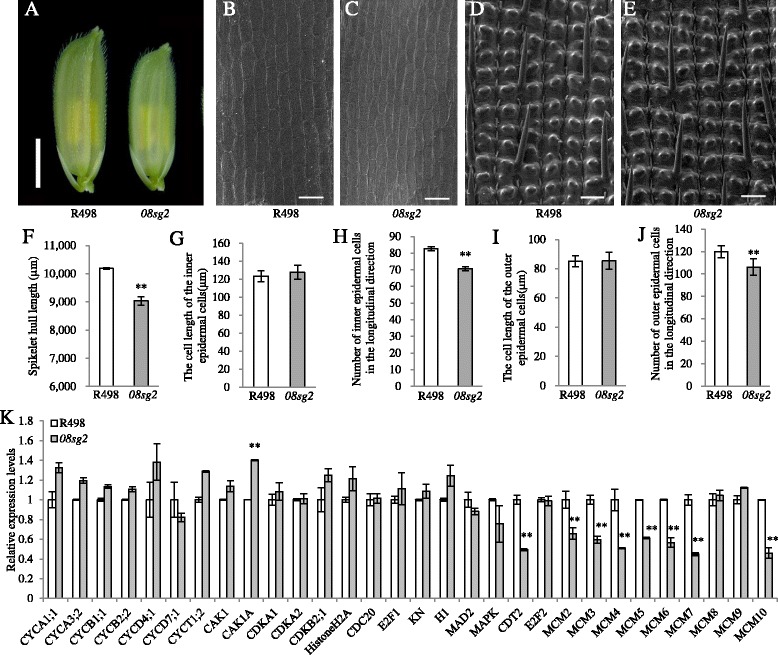
*08SG2* regulates grain length by changing cell division patterns. **a** The spikelet hulls of R498 and *08sg2* plants before anthesis. Scale bar, 3 mm. **b**, **c** Inner epidermal cells of the lemma observed by SEM. Scale bar, 100 μm. **d**, **e** Outer epidermal cells of the lemma observed by SEM. Scale bar, 100 μm. **f** Comparison of spikelet hull length between R498 and *08sg2* (*n* = 10). **g**, **h** Comparison analysis of the cell length, cell number in the inner epidermal cells (*n* = 30). **i**, **j** Comparison analysis of the cell length, cell number in the outer epidermal cells (*n* = 30). **k** Transcript levels of the cell cycle-related genes in R498 and *08sg2*. The analysis of relative expression levels was determined by qRT-PCR using 5–7 cm young panicles*. OsActin* was used as the control and the values of expression levels in R498 were set to one (*n* = 3). **f**-**k**, Data are given as means ± SD. Student’s t-test was used to generate the *P* values; ** indicate *P* < 0.01

To explore the cytological mechanism which led to the reduction of spikelet hulls, we used scanning electron microscopy (SEM) to compare the cell lengths of the inner and outer epidermal cells in spikelet hulls in R498 and *08sg2* (Fig. 2b-e). The cell length of the inner and outer epidermal cells of R498 and *08sg2* were not statistically different (Fig. 2g, i), but the number of inner and outer epidermal cells in the longitudinal direction was significantly less in *08sg2* than in R498 (Fig. 2h, j). Taken together, these results indicate that the reduction in cell number is responsible for the decreased grain length of *08sg2*. We then checked the expression level of cell-cycle related genes in R498 and *08sg2*. In young panicles, the expression level of *CDT2* and several *MCM* genes, which are involved in the G1-to-S transition, were significantly reduced in *08sg2* (Fig. 2k). These results suggest that *08SG2* regulates cell number by affecting cell proliferation.

Furthermore, we analyzed the expression level of several genes related to grain size. The expression of *TGW6*, which negatively controls grain length and thousand-grain weight (Ishimaru et al. 2013), was increased 1.8 times in *08sg2* (Additional file 1: Figure S7), consistent with the *08sg2* small grain phenotype. Therefore, *08SG2* is likely to regulate grain length through *TGW6*.

### *08sg2* is a loss of function mutant of *OsBAK1*

**Fig. 3:**
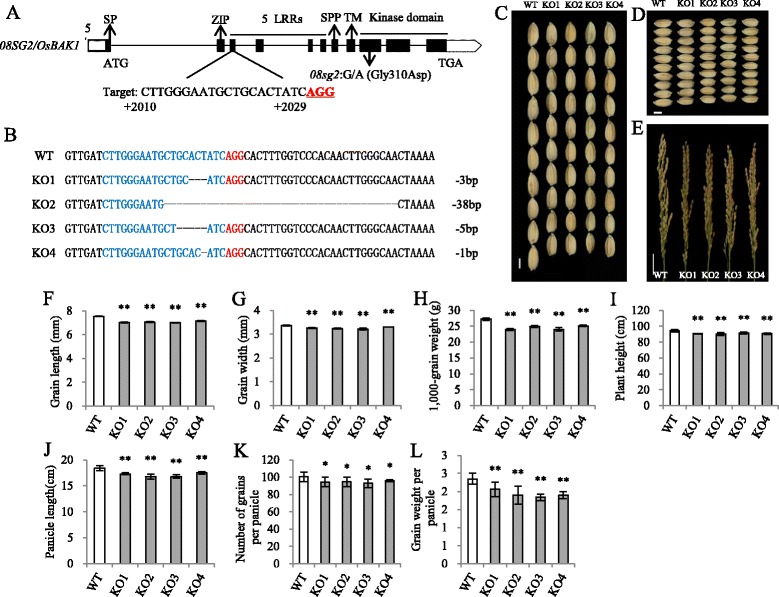
Generation of loss of function mutants of *OsBAK1* and comparisons of their phenotypes. **a** Schematic diagram of the target sites in the *OsBAK1*. The UTRs, exons and introns are indicated by blank rectangles, black rectangles and black lines, respectively. The targeted sites are labeled in black uppercase letters. and the protospacer adjacent motif (PAM) sequences are highlighted in red and underlined. The numbers labeled under the target sequences indicate the distance away from the translation start site (ATG, from 5’to 3^’^). SP, signal peptide. ZIP, leucine zipper. LRRs, leucine-rich repeats. SPP, pro-rich domain containing the Ser-Pro-Pro (SPP) motif. TM, transmembrane domain. The start codon (ATG) and the stop codon (TGA) are indicated. The *08sg2* mutation in the *OsBAK1* was shown. **b** Sequence alignment of the mutants within the *OsBAK1* target in the T_1_ plants. The wild-type (WT) sequence is depicted at the top with the target sequence in blue and the PAM sequence in red. The deleted sequences are shown by black hyphens, and the number of the deletions are showed on the right. **c**, **d** Comparisons of the grain length and grain width between WT and knock out mutants. Scale bar, 3 mm. **e** The panicle morphology of WT and knock-out mutants. Scale bar, 3 cm. **f** Grain length. **g** Grain width. **h** 1,000-grain weight. **i** Plant height. **j** Panicle length. **k** Number of grains per panicle. **l** Grain weight per panicle. All phenotypic data in F–L were measured from plants grown in paddies under normal cultivation conditions with three-time repeats. Data are given as means ± SD. Student’s t-test was used to generate the *P* values; *, ** indicate *P* < 0.05, *P* < 0.01, respectively

To further confirm that the mutation in *OsBAK1* was responsible for the *08sg2* phenotype, we used the CRISPR/Cas9 genome editing system (Shan et al. 2013) to knockout *OsBAK1* in *Nipponbare* (*japonica*) background. The target site was designed at the third exon of *OsBAK1* (Fig. 3a), ensuring a loss of function mutant. Four independent homozygous mutants (KO1-4) with different mutation types were obtained, and all mutations results in premature stop codons (Fig. 3b). Homozygous mutants of *OsBAK1* without T-DNA insertion were obtained from T_1_ generation. We then comprehensively investigated the agronomic traits in the T_2_. Relative to WT (*Nipponbare*), all four loss of function lines of *OsBAK1* were slightly shorter (Fig. 3i, Additional file 1: Figure S8), and the grain length and grain width decreased 6.3% and 3.4%, respectively (Fig. 3c, d, f, g), resulting a 10.1% reduction of 1000-grain weight (Fig. 3h). The panicle length and grain number per panicle were reduced 7.1% and 6.0%, respectively (Fig. 3e, j, k). Seed setting rate was not different (Additional file 1: Figure S9H). The reduction in both grain size and grain number resulted an 18.1% reduction of grain weight per panicle in all knockout mutants of *OsBAK1* (Fig. 3l). Together, *OsBAK1* knockout lines in the *japonica* background showed significantly decreased grain size and number, as in the *08sg2* mutant, but the reduction percentage was obvious smaller than *08sg2* in the *indica* background, except for grain width (Table 1), confirming that the SNP2 in *OsBAK1* is responsible for the *08sg2* mutant phenotype, and indicating that *08sg2* is a loss of function mutant but the effect of *08SG2/OsBAK1* on grain size and number is different between *indica* and *japonica* background.

**Table 1:** Comparisons of the reduction percentage of *08SG2/OsBAK1* mutation in *indica* and *japonica* backgrounds

	Grain length	Grain width	Plant height	Panicle length	Number of grains per panicle	Grain weight per panicle
*08sg2* vs R498	−10.2 ± 0.2	−2.7 ± 0.7	−8 ± 1.1	−11.9 ± 2.8	−9.7 ± 2.1	−23.3 ± 3.1
KO vs *Nipponbare*	−6.3 ± 0.6**	−3.4 ± 1.0	−3.8 ± 0.5**	−7.1 ± 1.7*	−6.0 ± 1.0*	−18.1 ± 3.5*

Reduction percentage (%) was calculated using the WT (R498 and *Nipponbare*, respectively) as control. KO indicated the average value of the four knock out mutants. Data are given as means ± SD. Student’s t-test was used to generate the *P* values; * and ** indicate *P* < 0.05 and *P* < 0.01, respectively

*OsBAK1* was expressed ubiquitously in all organs examined, with a relatively higher expression in developing young inflorescences (Additional file 1: Figure S10), supporting the biological roles of *08SG2*/*OsBAK1* in regulating grain size and grain number. The sequence alignment and phylogenetic analysis of the homologs of *OsBAK1* showed that *OsBAK1* was conserved in different species (Additional file 1: Figure S11; Additional file 1: Figure S12), notably, the amino acid substitution in *08sg2* (G310D), which was located in the kinase domain of *OsBAK1* (Fig. 3a), was highly conserved among different species (Additional file 1: Figure S11), suggesting that this amino acid may be important for *08SG2/OsBAK1* function.

### The *08sg2* mutant is insensitive to 24-epiBL

**Fig. 4:**
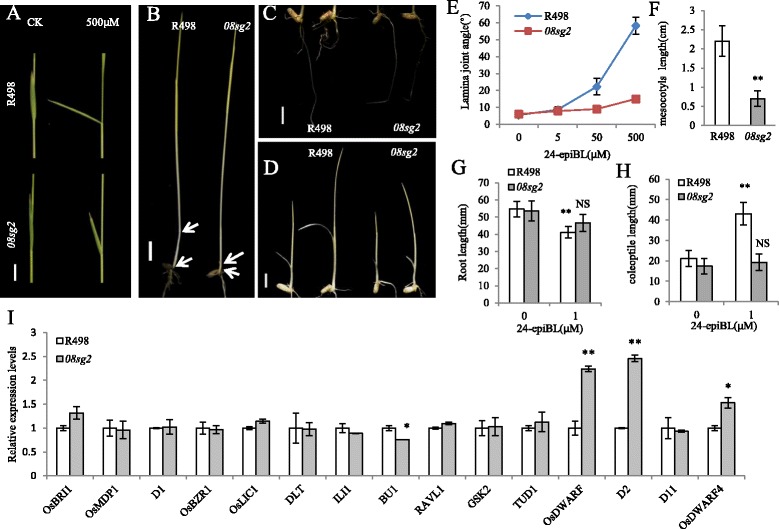
The *08sg2* mutant is insensitive to 24-epiBL. **a** Lamina joint of R498 and *08sg2* in response to 24-epiBL treatment. **b** Comparisons of the mesocotyls elongation between R498 and *08sg2* when plants were grown in complete darkness, arrows indicate the nodes. **c** Roots of R498 and *08sg2* seedlings grown on ½MS medium without (left) or with (right) 1 μM 24-epiBL treatment. **d** The coleoptile elongation of R498 and *08sg2* without (left) or with (right) 1 μM 24-epiBL treatment. **e** Measurements of the lamina joints angles of the wild type and *08sg2* after treatment with various concentrations of 24-epiBL. **f** The mesocotyls length of R498 and *08sg2* when plants were grown in complete darkness. **g** Roots length of R498 and *08sg2* seedlings grown on ½MS medium with 1 μM 24-epiBL treatment. **h** Coleoptile length of R498 and *08sg2* seedlings grown on ½MS medium with 1 μM 24-epiBL treatment. Bars = 10 mm. **i** The analysis of relative expression level was determined by qRT-PCR using young panicles (5-7 cm) during the inflorescence development. *OsActin* was used as the control and the values of expression levels in R498 were set to one (*n* = 3). Data are given as means ± SD. Student’s t-test was used to generate the *P* values; *, ** and NS indicate *P* < 0.05, *P* < 0.01 and no significant differences, respectively

*OsBAK1* was reported to participate in BR signal transduction, and its expression level affected sensitivity to BR (Li et al. 2009). Therefore, we performed a series of experiments to investigate the BR sensitivity of *08sg2*. Firstly, we analyzed the sensitivity of the *08sg2* mutant to 24-epibrassinolide (24-epiBL) with a lamina joint assay, which is a quantitative method to analyze response to BR (Yamamuro et al. 2000). The lamina joint angles of R498 increased significantly after 24-epiBL treatment, whereas those of the *08sg2* had no obvious response (Fig. 4a, e). The mesocotyl elongation of R498 was more obvious than that of *08sg2* when grown in darkness (Fig. 4b, f). In addition, many studies had indicated that BR would promote coleoptile growth and inhibit root elongation with wavy form in wild type plants, a good indicator of a BR-responsive phenotype (Duan et al. 2014; Jiang et al. 2012; Liu et al. 2015; Yamamuro et al. 2000). R498 and *08sg2* seeds were germinated and grown on ½MS medium with or without 1 μM 24-epiBL treatment, respectively. The roots of R498 exhibited a wavy, curly and reduced root length phenotype when treated with 1 μM 24-epiBL, while the *08sg2* showed similar growth patterns with or without exogenously supplied 24-epiBL (Fig. 4c, g). Furthermore, exogenous 24-epiBL significantly promoted wild type (R498) coleoptile growth, but there was no obvious promotion in *08sg2* (Fig. 4d, h). Together, these results suggest that the *08sg2* mutant is insensitive to BR.

### *08sg2* affects the expression of BR-related genes

*08SG2/OsBAK1* was involved in BR signal transduction (Li et al. 2009) and the *08sg2* mutant appeared insensitive to BR (Fig. 4a-h). We therefore analyzed the expression level of BR signaling-related genes, including *OsBRI1*, *OsMDP1*, *D1*, *OsBZR1*, *OsLIC1*, *DLT*, *ILI1*, *BU1*, *RAVL1*, *GSK2* and *TUD1* (Bai et al. 2007; Duan et al. 2006; Hu et al. 2013; Je et al. 2010; Tanaka et al. 2009; Tong et al. 2009; Tong et al. 2012; Wang et al. 2008; Wang et al. 2006; Yamamuro et al. 2000; Zhang et al. 2009). Only the expression of *BU1*, which was reported to be a positive regulator of BR signaling, was down-regulated in *08sg2* (Fig. 4i).

In addition, many studies had reported that BR mutants usually had feedback regulation of BR biosynthetic genes (Duan et al. 2014; Fang et al. 2016; Hong et al. 2003; Liu et al. 2015; Yamamuro et al. 2000). We therefore analyzed the expression of BR biosynthetic genes in R498 and *08sg2* in young panicles, including *OsDWARF*, *D2*, *D11*, *OsDWARF4* (Hong et al. 2003; Mori et al. 2002; Sakamoto et al. 2006; Tanabe et al. 2005). The expression levels of *OsDWARF*, *D2* and *OsDWARF4* were significantly up-regulated in *08sg2* (Fig. 4i). These results suggest that mutation in *08SG2/OsBAK1* results in the feedback regulation of BR biosynthesis in rice.

### *08SG2*/*OsBAK1* and *GS3* regulate grain length in independent pathways

**Fig. 5:**
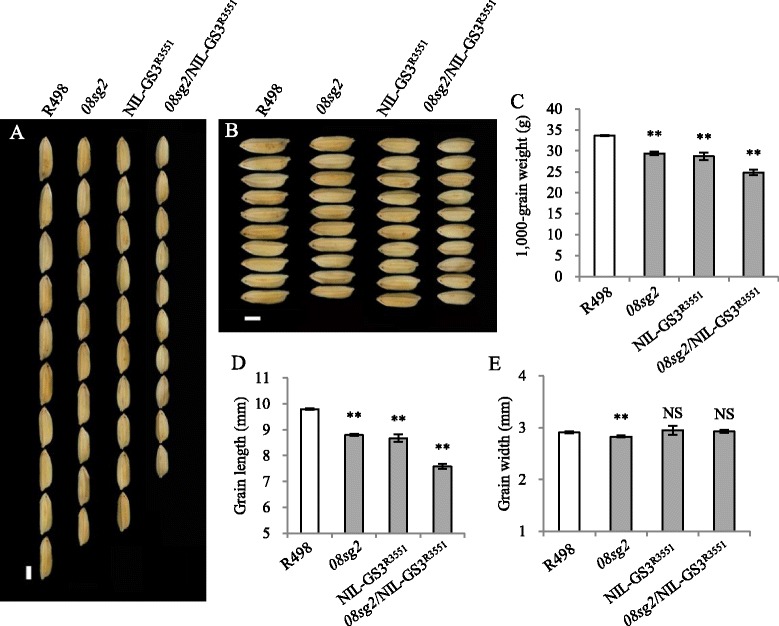
*08SG2*/*OsBAK1* and *GS3* regulate grain length in independent pathways. **a**, **b** Grain length and grain width performance in different plants, respectively. **c**-**e** The data statistical analysis of 1,000-grain weight, grain length, grain width in different plants, respectively. Data are given as means ± SD. Student’s t-test was used to generate the *P* values; ** and NS indicate *P* < 0.01 and no significant differences, respectively

The major grain length QTL *GS3* was reported to encode a protein with a transmembrane domain (Fan et al. 2006; Mao et al. 2010), and another study indicated that *GS3* might participate in BR signaling by indirectly influencing *BRS1* (Gao et al. 2015), which is involved in a *BRI1*-mediated BR signaling pathway in *Arabidopsis thaliana* (Li et al. 2001). Therefore, *08SG2/OsBAK1* might regulate grain length through *GS3*. The *gs3* in the *08sg2* background (R498) is loss of function, which was identified by a PCR-based marker based on the functional nucleotide polymorphism in *GS3* (Ramkumar et al. 2010), and exhibited a long-grain phenotype (Fig. 5a, Additional file 1: Figure S13B, D). We generated a near-isogenic line NIL-*GS3* in the same background with *08sg2*, using an *indica* rice variety R3551 as the donor of functional *GS3* (Additional file 1: Figure S13B, D). Compared with the grain size of R498, the grain length and width of NIL-*GS3* ^R3551^ was decreased by 11.5%, and increased 1.4%, respectively (Fig. 5a, b, d, e, Additional file 1: Figure S13B, C), consistent with the role of *GS3* as a major QTL for grain length and a minor QTL for grain width (Fan et al. 2006). We crossed *08sg2* with NIL-*GS3* ^R3551^ and generated a pyramiding line *08sg2*/NIL-*GS3* ^R3551^. The *08sg2*/NIL-*GS3* ^R3551^ plants showed a more severely reduced grain size; grain length and width were reduced 22.7%, and increased 0.7%, respectively, indicating that the effect of *08sg2*/NIL-*GS3* ^R3551^ on grain length was just a pyramiding effect of *08sg2/Osbak1* (−10.2%) and *GS3* (−11.5%) (Fig. 5). In addition, the *08SG2/OsBAK1* expression level was not affected in NIL-*GS3* panicle, nor was the *GS3* expression level in the *08sg2* mutant (Additional file 1: Figure S14). The effect of *08sg2*/NIL-*GS3* ^R3551^ on grain width (0.7%) was not a simple effect pyramiding of *08sg2/Osbak1* (−2.7%) and *GS3* (1.4%). Taken together, our results indicate that *08SG2/OsBAK1* and *GS3* likely regulate grain length in independent pathways, but they might have some genetic interaction regarding grain width.

## Discussion

### *08SG2*/*OsBAK1* plays a crucial role in the regulation of grain size and number

*08SG2/OsBAK1*, also called *OsSERK1*, belongs to the Somatic Embryogenesis Receptor Kinases (SERKs) family in rice (Hu et al. 2005; Ito et al. 2005). As a SERK family gene, *OsBAK1* was proven to play a basic role in mediating somatic embryogenesis in rice, and might play wider roles in other organs (Hu et al. 2005; Ito et al. 2005). Overexpression and knockdown of *OsBAK1* showed that *OsBAK1* controlled plant architecture and was involved in BR signaling (Li et al. 2009; Shimin et al. 2014). But there was no comprehensive investigation of *OsBAK1* function on yield-related traits, such as grain size and number, except that overexpression and knockdown of *OsBAK1* showed decreased grain length and increased grain width, respectively, in the *ZH11* background (Li et al. 2009). However, another study showed that *OsBAK1* didn’t affect grain size in the *Kitaake* background (Shimin et al. 2014). In this study, we used loss of function mutants of *08SG2/OsBAK1* in the *indica* and *japonica* backgrounds to investigate its genetic effect on grain size and number, and also measured other agronomic traits such as plant height, panicle length and grain weight (Fig. 1). Our results showed that the grain length and width were decreased in both *indica* (*Shuhui498*) and *japonica* (*Nipponbare*) background (Fig. 1f, g; Fig. 3f, g). These findings, different with previous reports, might be explained by *ZH11* (*japonica*) and *Kitaake* (*japonica*) backgrounds, or because expression of *OsBAK1* was not completely silenced by RNAi. Also, the decreased grain number in *08sg2/Osbak1* suggested that *08SG2/OsBAK1* is essential for rice grain number in both the *indica* and *japonica* backgrounds (Fig. 1m; Fig. 3k), which may be due to the up-regulated expression level of two negative regulators of panicle branching *OsCLV1* and OsCLV2 (Additional file 1: Figure S6).

Many studies had indicated that BR-related genes regulated grain size through altering cell expansion or cell proliferation, for example: *D2* (Fang et al. 2016) and *D11* (Wu et al. 2016a) regulated grain size by promoting cell expansion; *XIAO* (Jiang et al. 2012), *SMG1* (Duan et al. 2014) and *DSG1* (Liu et al. 2015) regulated grain size by affecting cell proliferation through cell cycle genes. Our results indicated that *08SG2/OsBAK1* positively regulates the development of spikelet hull by affecting cell proliferation (Fig. 2h, j), consistent with this notion, the expression levels of some cell-cycle related genes were down-regulated in the *08sg2* mutant (Fig. 2k), and this result is consistent with the cytological mechanism in internodes in the *OsBAK1* overexpression plants (Li et al. 2009). Our results provide a new evidence that BR may control grain size via altering cell proliferation through cell cycle genes.

Even the loss of function of *08SG2/OsBAK1* showed obvious reduction on grain size and grain number in both *indica* (*Shuhui498*) and *japonica* (*Nipponbare*) backgrounds, the differences of effect on grain size and grain number were still observed at different background, for example: loss of function of *08sg2/Osbak1* led to a 10.2% or 6.3% decrease in grain length, and to 9.7% or 6.0% decrease in grain number (Table 1) in *indica* (*Shuhui498*) and *japonica* (*Nipponbare*) backgrounds, respectively. This indicates that the function of *08SG2/OsBAK1* on grain size and number is affected by genetic background.

### *08SG2/OsBAK1* regulates grain length independently with *GS3*

*08SG2/OsBAK1* positively regulated grain length in rice. However, *GS3* is a negative regulator of grain length (Fan et al. 2006). The SNP substitution in *08sg2/Osbak1* led to similar reductions in the grain length in the *gs3* and *GS3* background (reduced 10.20% or 12.57%, respectively), and the mutation of *GS3* also led to a comparable increase in the grain length in the *08sg2/Osbak1* and *08SG2/OsBAK1* backgrounds (increased 16.09% or 13.03%, respectively) (Fig. 5). In addition, the expression levels of *O8SG2/OsBAK1* and *GS3* were not affected by each other, suggesting that *08SG2/OsBAK1* and *GS3* regulate grain length in independent pathways, with no genetic interaction.

### The amino acid substitution in the kinase domain of *08SG2/OsBAK1* lead to loss function

The amino acid substitution (G310D) in *08sg2* is located in the kinase domain of *OsBAK1* and this glycine is highly conserved in other species (Fig. 3a, Additional file 1: Figure S11). This amino acid substitution leads a phenotype similar to the loss of function mutant (Fig. 1; Fig. 3). *OsBAK1* encodes a receptor-like protein kinase (RLK) (Ito et al. 2005). Both *OsBAK1* and its homolog *BAK1* in *Arabidopsis* are functional protein kinases capable of auto-phosphorylation, and a mutation at a conserved site (K329E, K330E, respectively) in the kinase domain led to loss of kinase activity (Li et al. 2002; Shimin et al. 2014). Several other studies in *Arabidopsis* showed that the amino acid substitution at a conserved site in the kinase domain of RLKs abolished kinase activity (Braun et al. 1997; Horn and Walker 1994), but the amino acid change in *08sg2* is different. Therefore, this substituted amino acid might play a role in protein function of *08SG2/OsBAK1*, and it is worthy to investigate whether this amino acid substitution affects protein kinase activity, or other molecular functions of *08SG2/OsBAK1*.

## Conclusions

*08SG2/OsBAK1* plays an essential role in the regulation of grain size, grain number and plant height in both the *indica* and *japonica* backgrounds, and is important for rice yield. The function of *08SG2/OsBAK1* on grain size and number is affected by genetic background. *08SG2/OsBAK1* regulates grain size by participating in cell proliferation, and is independent of *GS3*. The glycine substituted in *08sg2* is located in the kinase domain, is highly conserved among different species, and is presumably important for the molecular function of *08SG2/OsBAK1*. Together, our work is helpful for unveiling the molecular function of *08SG2/OsBAK1*, and indicating that *08SG2/OsBAK1* is a potential target to manipulate for increasing rice yield.

## Methods

### Plant materials and growth conditions

The *small grain* mutant (*sg2*) was initially identified from an ethyl methanesulfonate (EMS) mutant population of an *indica* variety *Shuhui498* (R498). *08sg2* was isolated from *sg2* in the segregation population by linkage marker and backcrossed with R498 three times. All plants were grown in paddies at the Rice Research Institute of Sichuan Agricultural University (Chengdu, China) and Hainan (Lingshui, China) under normal cultivation conditions.

### Cytological analysis by scanning electron microscopy (SEM)

Spikelet hulls from *08sg2* and R498 plants were collected before fertilization and fixed in 2.5% glutaraldehyde, which was prepared as described (Hu et al. 2015). The fixed samples were dehydrated in a graded ethanol series (from 30% to 100%, 100%), followed by substitution using isopentyl acetate. The critical-point Dryer (Quorum K850, England) was used to dry samples. The samples were sputter-coated with platinum using a magnetron sputtering apparatus (JS-1600, Beijing, China). The inner and outer epidermal cells of lemma of the spikelet hulls were observed by SEM (JSM-7500 F, JEOL, Japan). Cell lengths were measured using Image J software.

### Gene mapping

MutMap (Abe et al. 2012) was applied for gene mapping. Briefly, we generated an F_2_ population of a cross between the *sg2* mutant and the wild type (R498). DNA of 25 F_2_ progeny with small grains as *sg2* was extracted and bulked in an equal ratio, then this bulked DNA was subjected to whole-genome sequencing. DNA from R498 was re-sequenced as a control. The SNPs/INDELs indexes were calculated as previously described (Abe et al. 2012). We simultaneously measured allele segregation using Euclidean distance (ED) (Additional file 1: Figure S2B) as previously described (Hill et al. 2013).

### CRISPR/Cas9 plasmid construction, plant transformation and mutation detection

To obtain loss of function mutants, we designed the target site at the third exon of *08SG2/OsBAK1* (Fig. 3a), then generated the CRISPR/Cas9 plasmid construct using the Biogle CRISPR/Cas kit (BGK03, http://www.biogle.cn/) according to instructions. The Oligo sequence pair (F:5′-TGTGTGCTTGGGAATGCTGCACTATC-3′, R:5′-AAACGATAGTGCAGCATTCCCAAGCA-3′) was generated using the online system (BGK03, http://www.biogle.cn/index/excrispr). The final CRISPR/Cas9 plasmid construction pBGK03-*OsBAK1* was introduced into *Agrobacterium tumefaciens* strain EHA105, and transformation of *Nipponbare* was performed as previously described (Hiei et al. 1994). For mutation detection, genomic DNA was extracted from T_0_ transgenic plants and primer pairs flanking the designed target site were used for PCR amplification. PCR products were sequenced to obtain knockout plants. The sequencing primers are listed in Additional file 2: Table S3.

### Brassinosteroid sensitivity assay

For lamina joint inclination analysis, after soaking and pregermination, the uniform germinated seeds of R498 and *08sg2* were sown in soil and cultured in the plant growth chamber at 28 °C until two-leaf stage. 1 μl of ethanol solution containing various concentrations (0, 5, 50, 500 μM) of 24-epiBL were dropped at the lamina joints of the second leaf of R498 and *08sg2*. After cultured for 3 days, the angles between the lamina and its leaf sheath were photographed and measured. 30 plants were measured for each treatment with three replications.

For the mesocotyl elongation assay, after soaking and pre-germination, the uniform germinated seeds of R498 and *08sg2* were sown in soil and cultured in the plant growth chamber at 28 °C in complete darkness for about 12 days. The mesocotyls of R498 and *08sg2* were photographed and measured. 30 plants were measured for each treatment with three replications.

For the root elongation inhibition and coleoptile elongation assays, seeds of R498 and *08sg2* were dehusked, and sterilized by washing the seeds as follows: 75% ethanol for 1 min, sterilized water for five times, 2% NaClO for 30 min, then rinsed several times with sterilized water. The seeds were then germinated on half-strength Murashige and Skoog medium supplemented with 0, lμM 24-epiBL at 28 °C. About a week later, root and coleoptile were photographed and lengths measured. 30 plants were measured for each treatment with three replications.

### RNA extraction, cDNA synthesis and quantitative real-time RT-PCR

Total RNA was extracted from young panicles of R498, *08sg2*, NIL-*GS3* ^R3551^ using Plant RNA Kit according to the product manual (OMEGA). To obtain cDNA, about 500 ng of the total RNA was used for a genomic DNA elimination reaction and a reverse-transcription reaction using PrimeScript^TM^ RT Reagent Kit with gDNA Eraser according to the product manual (TaKaRa). Quantitative real-time RT-PCR analysis was performed on the CFX96^TM^ Real-Time PCR system (Bio-Rad) with KAPA SYBR® FAST qPCR Kit (KAPA). Actin was used as an internal control in all analyses. Three biological replicates were performed for each gene. The primers used in quantitative real-time RT-PCR are listed in Additional file 2: Table S4.

## Additional files

Additional file 1: Figure S1. Morphology of wild type (R498) and *sg2* mutant. **Figure S2.** Identification of the causal SNP of the *small grain* (*sg2*) mutant using MutMap approach. **Figure S3.** Linkage analysis of *08sg2*. **Figure S4.** The *08sg2* mutant exhibited slightly shorter plant height because of the reduced panicle and the uppermost internode. **Figure S5.** Effect of *08SG2* on endosperm size and grain filling. **Figure S6.** Comparisons of transcripts of genes determining panicle branching in R498 and *08sg2*. **Figure S7.** Comparisons of transcripts of genes determining grain size in R498 and *08sg2*. **Figure S8.** Comparisons of phenotypes of wild type (WT, *Nipponbare*) and knock out (KO) mutants at reproductive stage. **Figure S9.** Data statistics of the other agronomic traits in R498 and *08sg2*, the wild type (WT) and knock-out (KO) mutants. **Figure S10.** The expression pattern of *08SG2/OsBAK1* in different tissues. **Figure S11.** Sequence alignment of *08SG2/OsBAK1* and its orthologs in plants. **Figure S12.** Phylogenetic and protein similarity analysis of *08SG2/OsBAK1* orthologs in plants. **Figure S13.** The donor parent of *GS3* has significantly smaller grain size than R498. **Figure S14.** *08SG2* and *GS3* don’t interact at transcriptional level. **Table S1.** Agronomic traits of R498 and *sg2.* **Table S2.** A cluster of three SNPs with SNP index of 1 on chromosome 8 (PPTX 2792 kb)

Additional file 2: Table S3. Primers used for PCR detection. **Table S4.** Primers used for qPCR analysis (XLS 58 kb)
