# Supplementary material for: 08SG2/OsBAK1 regulates grain size and number, and functions differently in Indica and Japonica backgrounds in rice
Source: Rice (N Y). 2017 May 25;10:25. doi: 10.1186/s12284-017-0165-2 (PMC5445040; doi:10.1186/s12284-017-0165-2)
Supplement: Supplementary file 1 — Morphology of wild type (R498) and sg2 mutant. Figure S2. Identification of the causal SNP of the small grain (sg2) mutant using MutMap approach. Figure S3. Linkage analysis of 08sg2. Figure S4. The 08sg2 mutant exhibited slightly shorter plant height because of the reduced panicle and the uppermost internode. Figure S5. Effect of 08SG2 on endosperm size and grain filling. Figure S6. Comparisons of transcripts of genes determining panicle branching in R498 and 08sg2. Figure S7. Comparisons of transcripts of genes determining grain size in R498 and 08sg2. Figure S8. Comparisons of phenotypes of wild type (WT, Nipponbare) and knock out (KO) mutants at reproductive stage. Figure S9. Data statistics of the other agronomic traits in R498 and 08sg2, the wild type (WT) and knock-out (KO) mutants. Figure S10. The expression pattern of 08SG2/OsBAK1 in different tissues. Figure S11. Sequence alignment of 08SG2/OsBAK1 and its orthologs in plants. Figure S12. Phylogenetic and protein similarity analysis of 08SG2/OsBAK1 orthologs in plants. Figure S13. The donor parent of GS3 has significantly smaller grain size than R498. Figure S14. 08SG2 and GS3 don’t interact at transcriptional level. Table S1. Agronomic traits of R498 and sg2. Table S2. A cluster of three SNPs with SNP index of 1 on chromosome 8 (PPTX 2792 kb) [file 12284_2017_165_MOESM1_ESM.pptx]

## Slide 1
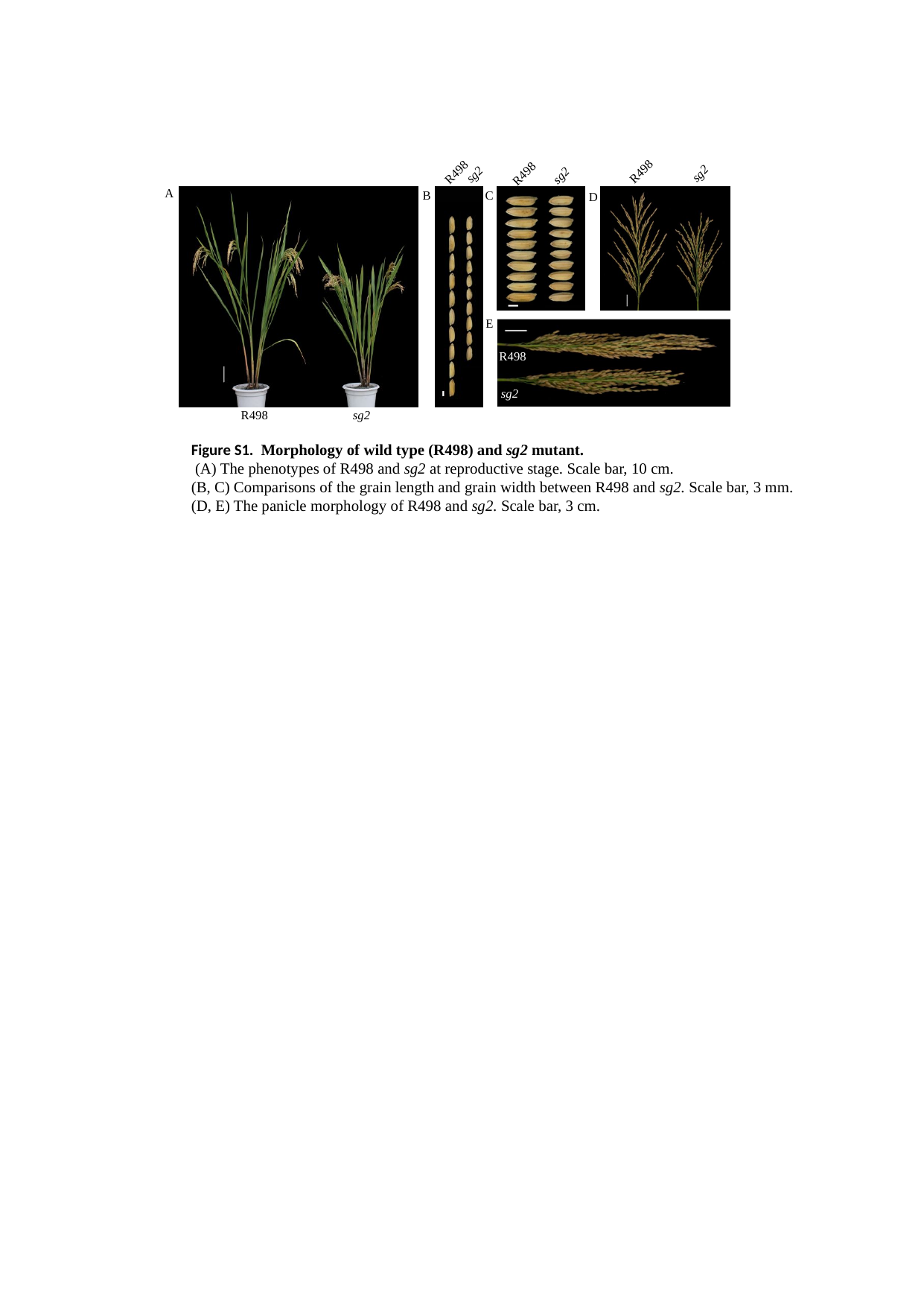

sg2
sg2
R498
R498
sg2
R498
A
C
B
D
E
R498
sg2
R498
sg2
Figure S1. Morphology of wild type (R498) and sg2 mutant.
 (A) The phenotypes of R498 and sg2 at reproductive stage. Scale bar, 10 cm.
(B, C) Comparisons of the grain length and grain width between R498 and sg2. Scale bar, 3 mm.
(D, E) The panicle morphology of R498 and sg2. Scale bar, 3 cm.

## Slide 2
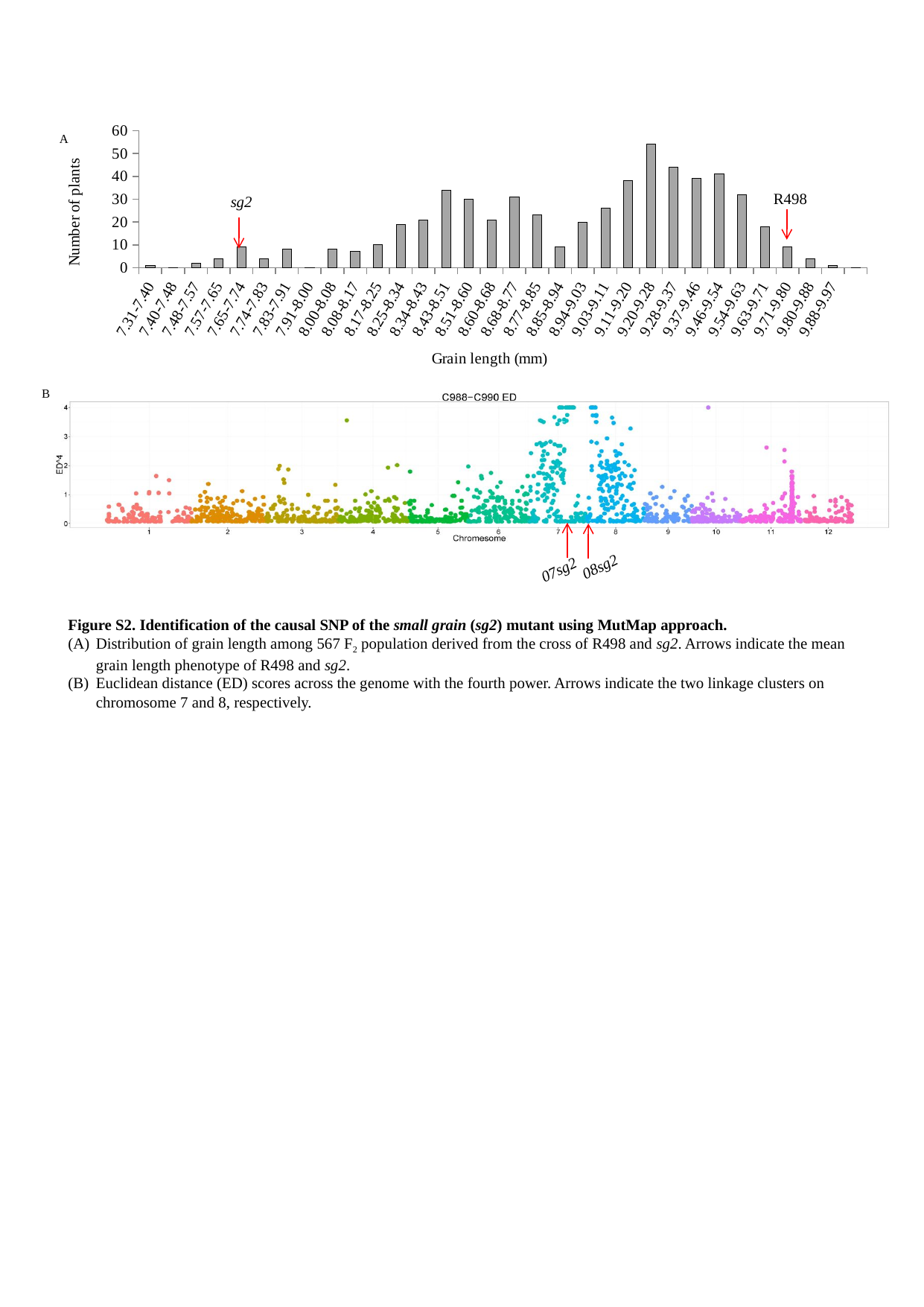

### Chart
| Category | |
|---|---|
| 7.31-7.40 | 1.0 |
| 7.40-7.48 | 0.0 |
| 7.48-7.57 | 2.0 |
| 7.57-7.65 | 4.0 |
| 7.65-7.74 | 9.0 |
| 7.74-7.83 | 4.0 |
| 7.83-7.91 | 8.0 |
| 7.91-8.00 | 0.0 |
| 8.00-8.08 | 8.0 |
| 8.08-8.17 | 7.0 |
| 8.17-8.25 | 10.0 |
| 8.25-8.34 | 19.0 |
| 8.34-8.43 | 21.0 |
| 8.43-8.51 | 34.0 |
| 8.51-8.60 | 30.0 |
| 8.60-8.68 | 21.0 |
| 8.68-8.77 | 31.0 |
| 8.77-8.85 | 23.0 |
| 8.85-8.94 | 9.0 |
| 8.94-9.03 | 20.0 |
| 9.03-9.11 | 26.0 |
| 9.11-9.20 | 38.0 |
| 9.20-9.28 | 54.0 |
| 9.28-9.37 | 44.0 |
| 9.37-9.46 | 39.0 |
| 9.46-9.54 | 41.0 |
| 9.54-9.63 | 32.0 |
| 9.63-9.71 | 18.0 |
| 9.71-9.80 | 9.0 |
| 9.80-9.88 | 4.0 |
| 9.88-9.97 | 1.0 |R498
sg2
A
B
08sg2
07sg2
Figure S2. Identification of the causal SNP of the small grain (sg2) mutant using MutMap approach.
Distribution of grain length among 567 F2 population derived from the cross of R498 and sg2. Arrows indicate the mean grain length phenotype of R498 and sg2.
Euclidean distance (ED) scores across the genome with the fourth power. Arrows indicate the two linkage clusters on chromosome 7 and 8, respectively.

## Slide 3
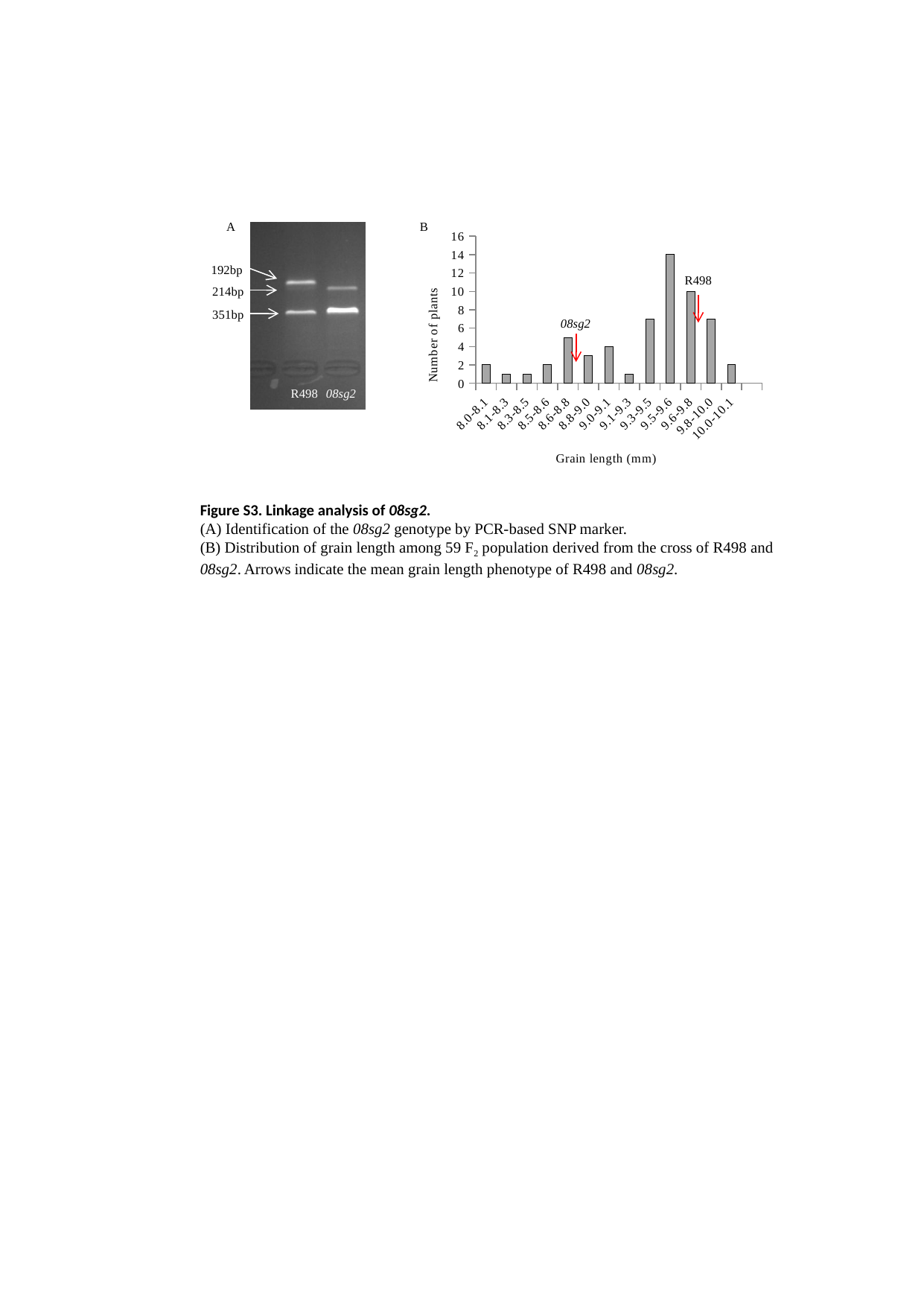

A
B
### Chart
| Category | |
|---|---|
| 8.0-8.1 | 2.0 |
| 8.1-8.3 | 1.0 |
| 8.3-8.5 | 1.0 |
| 8.5-8.6 | 2.0 |
| 8.6-8.8 | 5.0 |
| 8.8-9.0 | 3.0 |
| 9.0-9.1 | 4.0 |
| 9.1-9.3 | 1.0 |
| 9.3-9.5 | 7.0 |
| 9.5-9.6 | 14.0 |
| 9.6-9.8 | 10.0 |
| 9.8-10.0 | 7.0 |
| 10.0-10.1 | 2.0 |R498
08sg2
192bp
214bp
351bp
R498
08sg2
Figure S3. Linkage analysis of 08sg2.
(A) Identification of the 08sg2 genotype by PCR-based SNP marker.
(B) Distribution of grain length among 59 F2 population derived from the cross of R498 and 08sg2. Arrows indicate the mean grain length phenotype of R498 and 08sg2.

## Slide 4
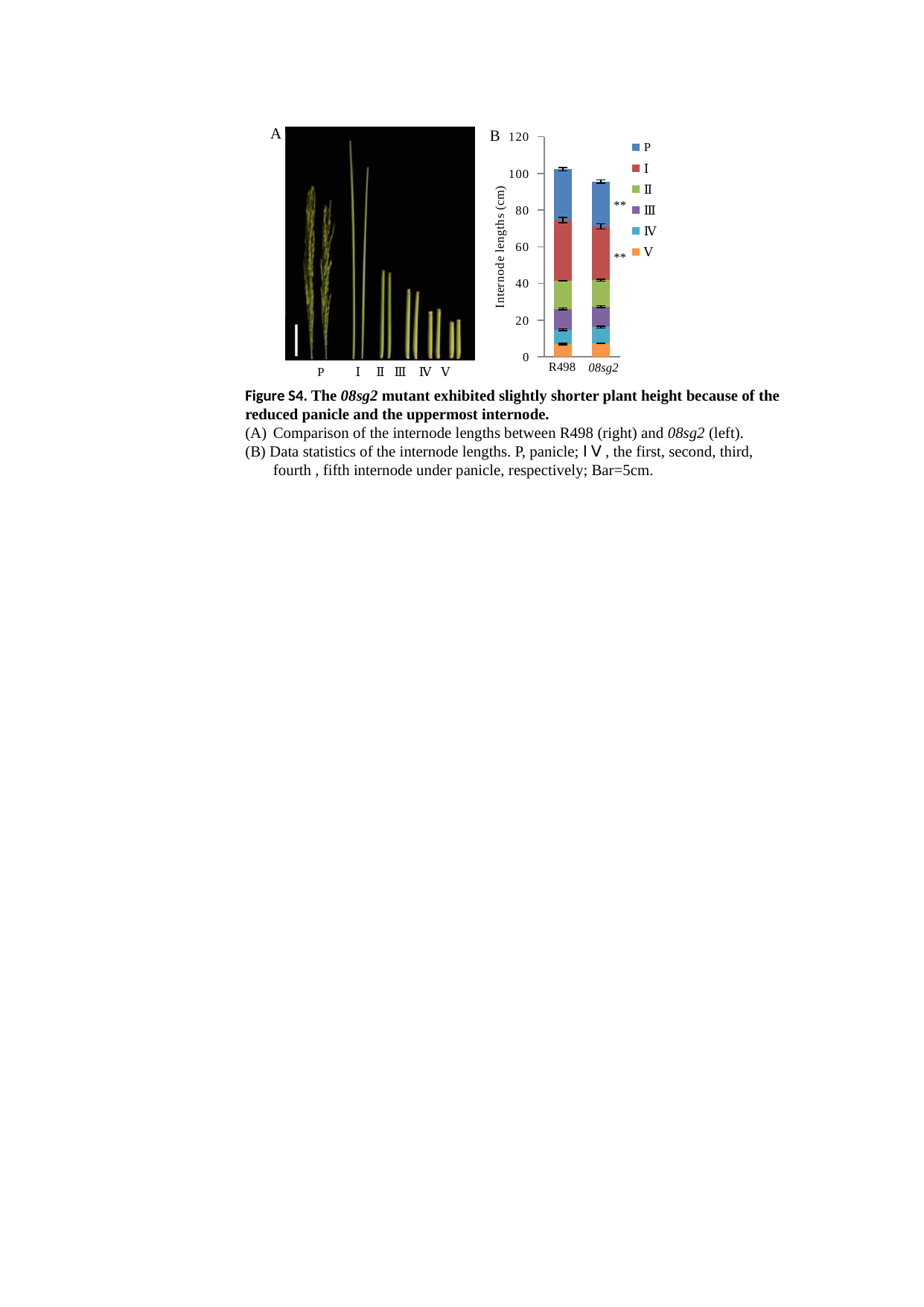

A
B
### Chart
| Category | Ⅴ | Ⅳ | Ⅲ | Ⅱ | Ⅰ | P |
|---|---|---|---|---|---|---|
| R498 | 6.9666666666666694 | 7.760000000000001 | 11.461538461538462 | 15.164285714285716 | 33.20000000000001 | 27.7 |
| 08sg2 | 7.4 | 8.733333333333318 | 11.186666666666676 | 14.446666666666674 | 29.3 | 24.6 |**
**
R498
08sg2
 P Ⅰ Ⅱ Ⅲ Ⅳ Ⅴ
Figure S4. The 08sg2 mutant exhibited slightly shorter plant height because of the reduced panicle and the uppermost internode.
Comparison of the internode lengths between R498 (right) and 08sg2 (left).
(B) Data statistics of the internode lengths. P, panicle; Ⅰ－Ⅴ, the first, second, third, fourth , fifth internode under panicle, respectively; Bar=5cm.

## Slide 5
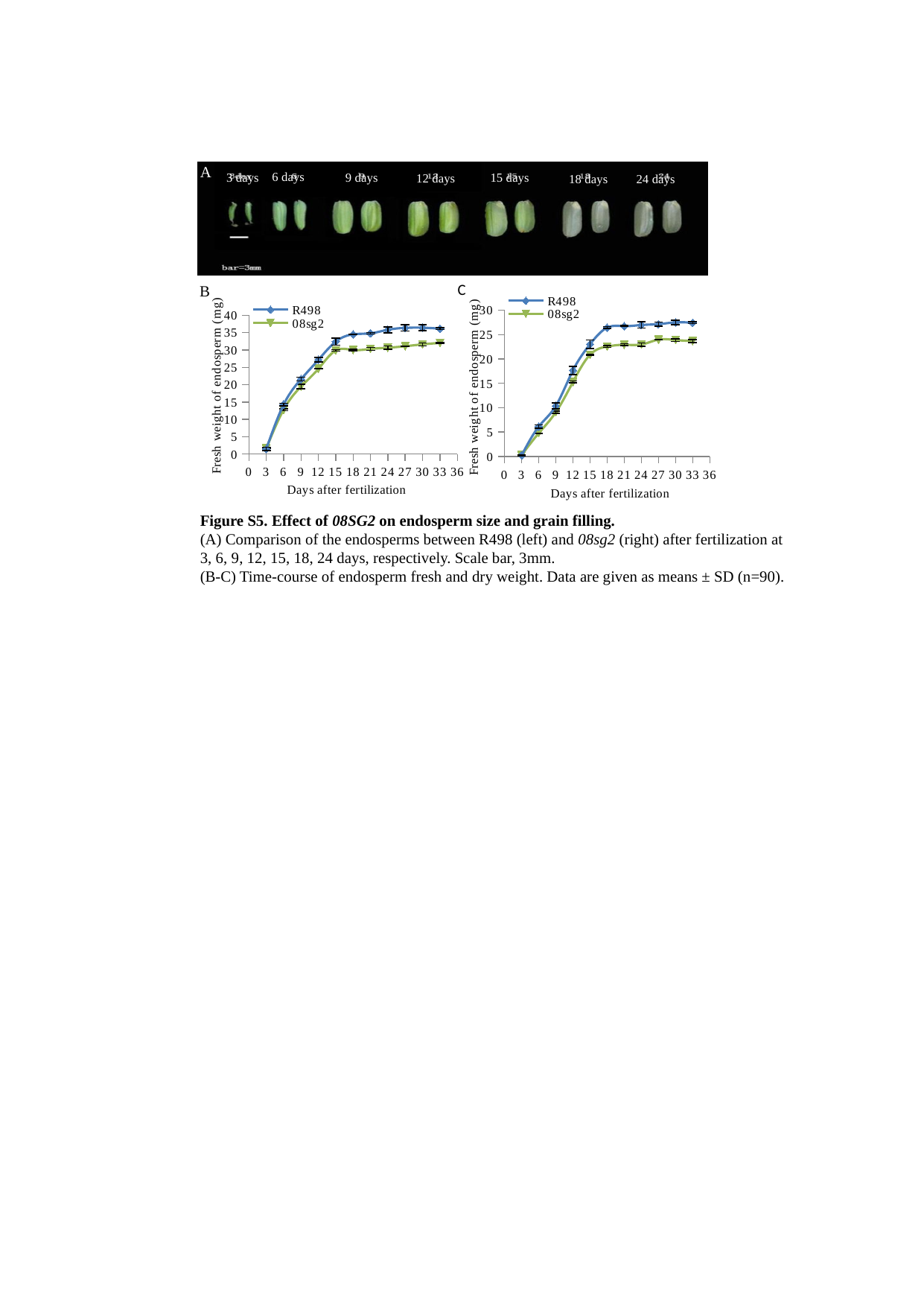

A
6 days
3 days
9 days
15 days
12 days
18 days
24 days
C
B
### Chart
| Category | R498 | 08sg2 |
|---|---|---|
### Chart
| Category | R498 | 08sg2 |
|---|---|---|Figure S5. Effect of 08SG2 on endosperm size and grain filling.
(A) Comparison of the endosperms between R498 (left) and 08sg2 (right) after fertilization at 3, 6, 9, 12, 15, 18, 24 days, respectively. Scale bar, 3mm.
(B-C) Time-course of endosperm fresh and dry weight. Data are given as means ± SD (n=90).

## Slide 6
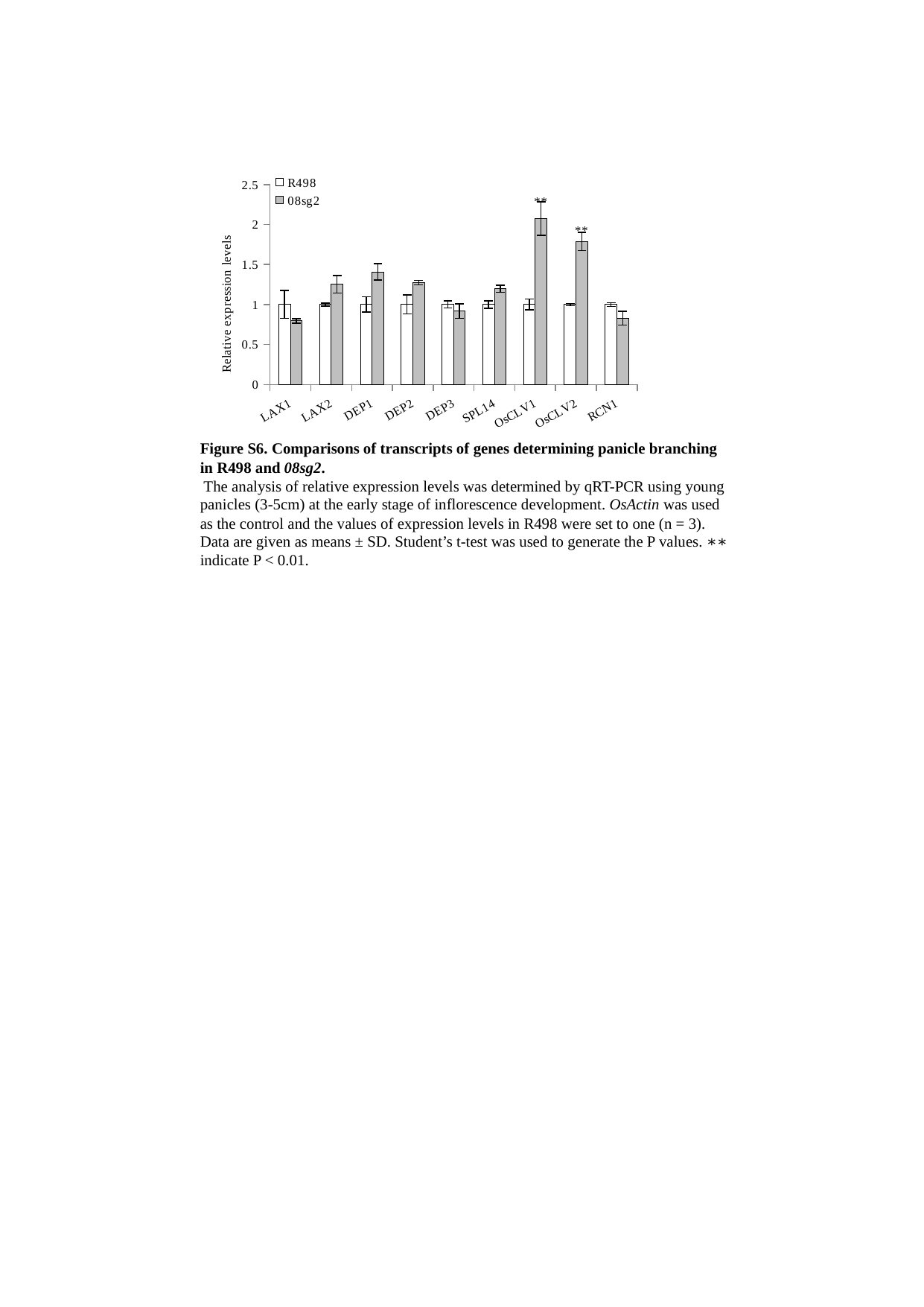

### Chart
| Category | R498 | 08sg2 |
|---|---|---|
| LAX1 | 1.0 | 0.7941783349005826 |
| LAX2 | 1.0 | 1.2522962610409298 |
| DEP1 | 1.0 | 1.4087098564321356 |
| DEP2 | 1.0 | 1.2746680613038661 |
| DEP3 | 1.0 | 0.9180418726396625 |
| SPL14 | 1.0 | 1.1964947874225798 |
| OsCLV1 | 1.0 | 2.07518132736332 |
| OsCLV2 | 1.0 | 1.7880459858525501 |
| RCN1 | 1.0 | 0.8304407206944953 |Figure S6. Comparisons of transcripts of genes determining panicle branching in R498 and 08sg2.
 The analysis of relative expression levels was determined by qRT-PCR using young panicles (3-5cm) at the early stage of inflorescence development. OsActin was used as the control and the values of expression levels in R498 were set to one (n = 3). Data are given as means ± SD. Student’s t-test was used to generate the P values. ∗∗ indicate P < 0.01.

## Slide 7
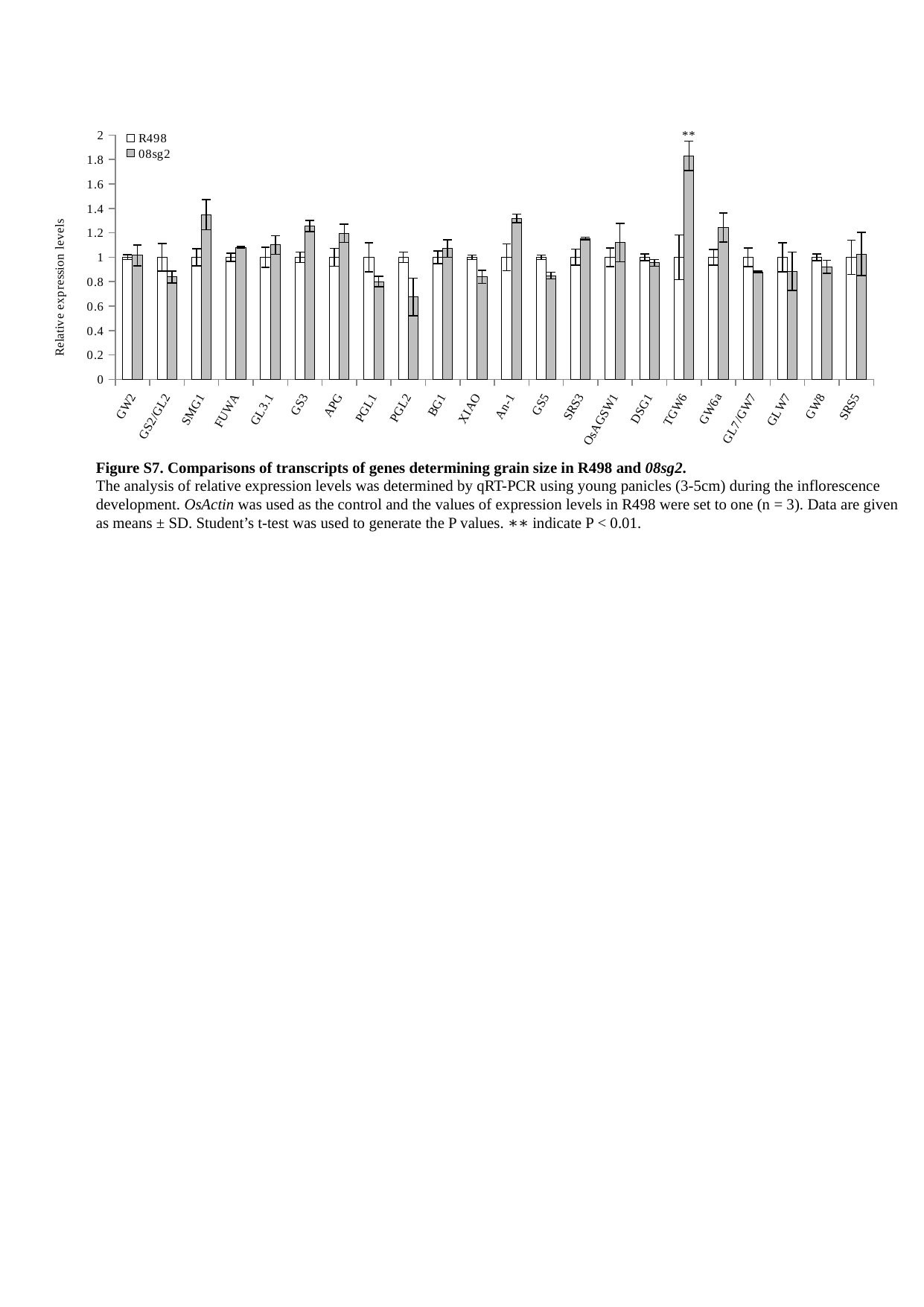

### Chart
| Category | R498 | 08sg2 |
|---|---|---|
| GW2 | 1.0 | 1.015567285830983 |
| GS2/GL2 | 1.0 | 0.8386200157525792 |
| SMG1 | 1.0 | 1.3487299407603797 |
| FUWA | 1.0 | 1.080180894277176 |
| GL3.1 | 1.0 | 1.1005536822259518 |
| GS3 | 1.0 | 1.255477161644187 |
| APG | 1.0 | 1.1966165781059943 |
| PGL1 | 1.0 | 0.8015095387847505 |
| PGL2 | 1.0 | 0.6756766501864525 |
| BG1 | 1.0 | 1.071098562628337 |
| XIAO | 1.0 | 0.8395751009674386 |
| An-1 | 1.0 | 1.3193059990559268 |
| GS5 | 1.0 | 0.850050641270934 |
| SRS3 | 1.0 | 1.1532472213539715 |
| OsAGSW1 | 1.0 | 1.121041080336966 |
| DSG1 | 1.0 | 0.954006717708496 |
| TGW6 | 1.0 | 1.8298786291321438 |
| GW6a | 1.0 | 1.243431596201553 |
| GL7/GW7 | 1.0 | 0.8802262697620943 |
| GLW7 | 1.0 | 0.8859313337899324 |
| GW8 | 1.0 | 0.9214614869549995 |
| SRS5 | 1.0 | 1.0269637182904139 |Figure S7. Comparisons of transcripts of genes determining grain size in R498 and 08sg2.
The analysis of relative expression levels was determined by qRT-PCR using young panicles (3-5cm) during the inflorescence development. OsActin was used as the control and the values of expression levels in R498 were set to one (n = 3). Data are given as means ± SD. Student’s t-test was used to generate the P values. ∗∗ indicate P < 0.01.

## Slide 8
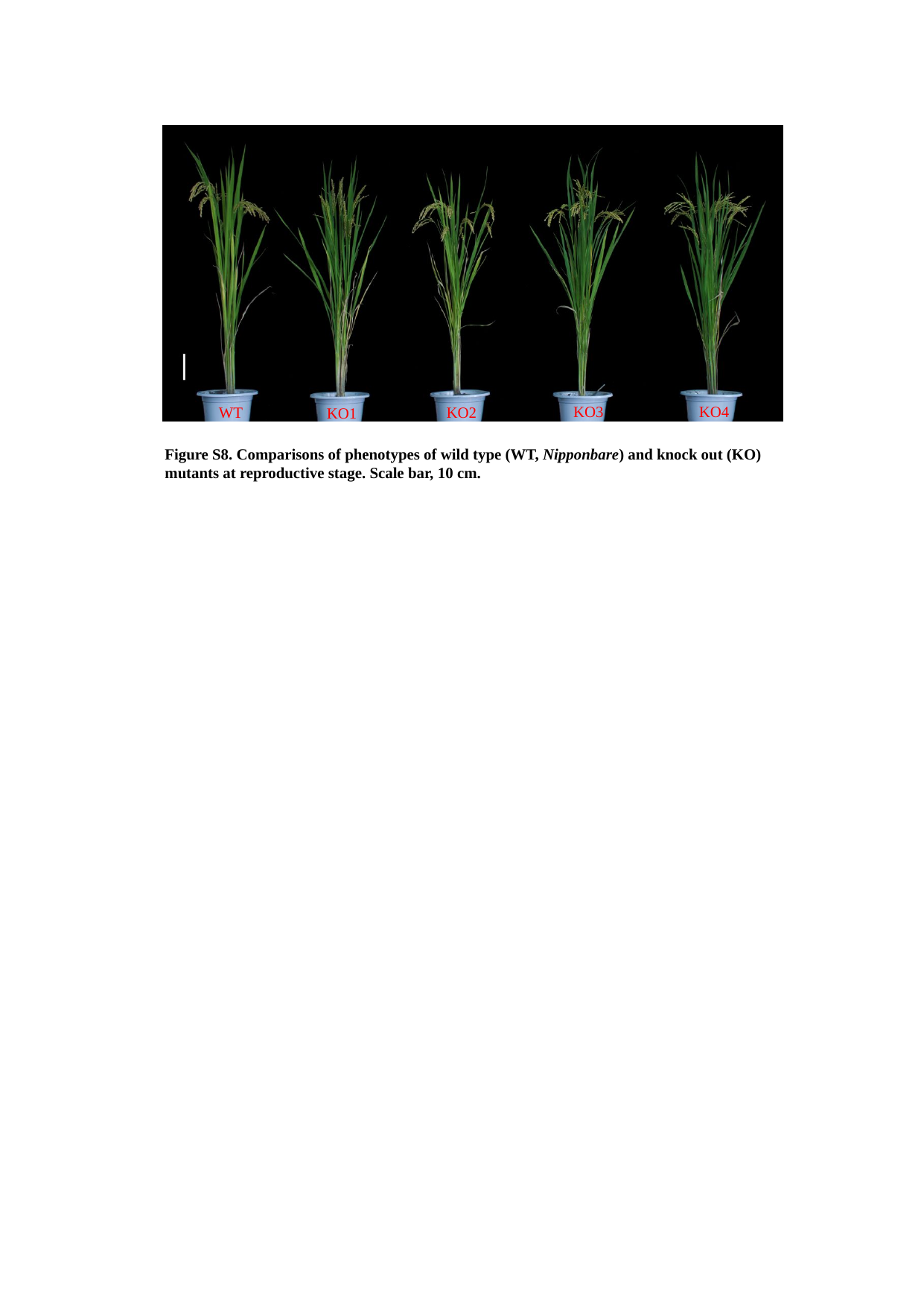

KO3
KO4
KO2
WT
KO1
Figure S8. Comparisons of phenotypes of wild type (WT, Nipponbare) and knock out (KO) mutants at reproductive stage. Scale bar, 10 cm.

## Slide 9
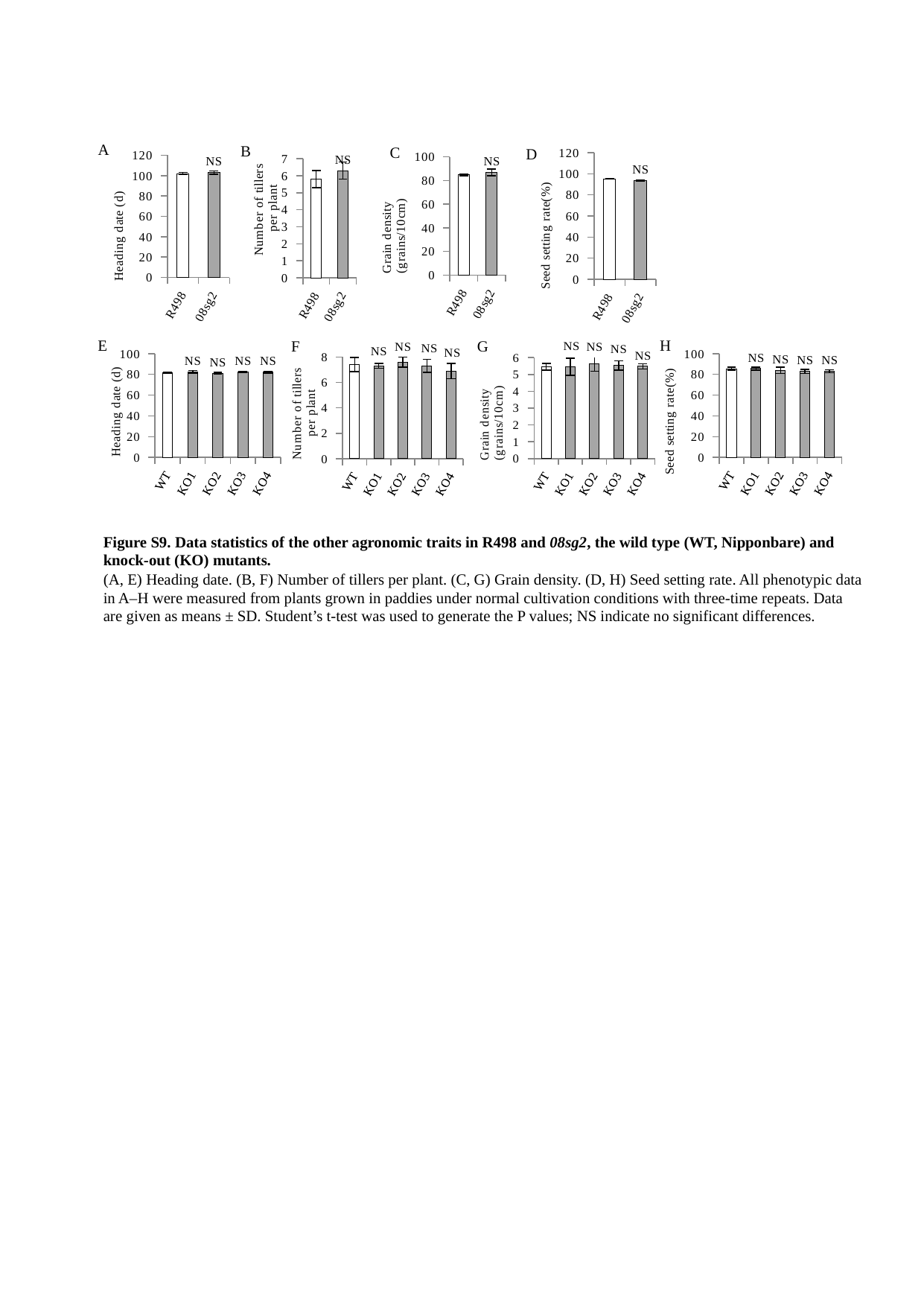

A
B
C
D
### Chart
| Category | |
|---|---|
| R498 | 5.8 |
| 08sg2 | 6.3 |
### Chart
| Category | |
|---|---|
| R498 | 84.9 |
| 08sg2 | 86.9 |
### Chart
| Category | |
|---|---|
| R498 | 95.3 |
| 08sg2 | 93.5 |
### Chart
| Category | |
|---|---|
| R498 | 102.0 |
| 08sg2 | 103.0 |H
E
G
F
### Chart
| Category | |
|---|---|
| WT | 85.46000000000002 |
| KO1 | 85.54 |
| KO2 | 83.9 |
| KO3 | 82.77 |
| KO4 | 82.85 |
### Chart
| Category | |
|---|---|
| WT | 81.66666666666667 |
| KO1 | 82.33333333333326 |
| KO2 | 81.0 |
| KO3 | 82.33333333333326 |
| KO4 | 82.0 |
### Chart
| Category | |
|---|---|
| WT | 5.46 |
| KO1 | 5.46 |
| KO2 | 5.63 |
| KO3 | 5.53 |
| KO4 | 5.48 |
### Chart
| Category | |
|---|---|
| WT | 7.4 |
| KO1 | 7.3 |
| KO2 | 7.6 |
| KO3 | 7.3 |
| KO4 | 6.9 |Figure S9. Data statistics of the other agronomic traits in R498 and 08sg2, the wild type (WT, Nipponbare) and knock-out (KO) mutants.
(A, E) Heading date. (B, F) Number of tillers per plant. (C, G) Grain density. (D, H) Seed setting rate. All phenotypic data in A–H were measured from plants grown in paddies under normal cultivation conditions with three-time repeats. Data are given as means ± SD. Student’s t-test was used to generate the P values; NS indicate no significant differences.

## Slide 10
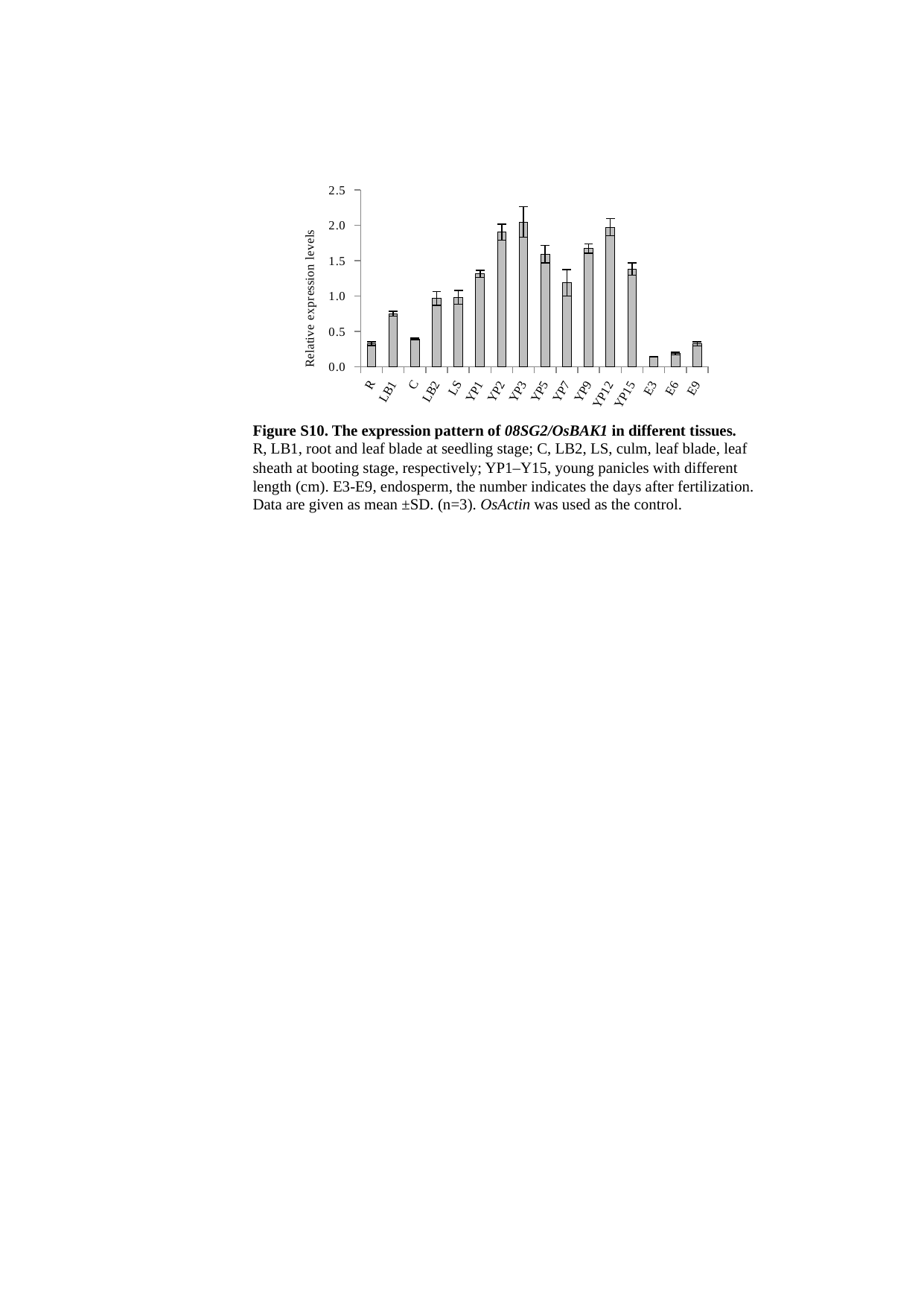

### Chart
| Category | Expression |
|---|---|
| R | 0.3242277642097138 |
| LB1 | 0.7475597388188386 |
| C | 0.39181462265167555 |
| LB2 | 0.9640438904523256 |
| LS | 0.9807835020676076 |
| YP1 | 1.3131859736305862 |
| YP2 | 1.9037596663891299 |
| YP3 | 2.044590107227861 |
| YP5 | 1.5897193259726499 |
| YP7 | 1.18535124668336 |
| YP9 | 1.6680837084677367 |
| YP12 | 1.9714908520025098 |
| YP15 | 1.3797347361005499 |
| E3 | 0.14094312807721499 |
| E6 | 0.18424971130146542 |
| E9 | 0.322767137836795 |Figure S10. The expression pattern of 08SG2/OsBAK1 in different tissues.
R, LB1, root and leaf blade at seedling stage; C, LB2, LS, culm, leaf blade, leaf sheath at booting stage, respectively; YP1–Y15, young panicles with different length (cm). E3-E9, endosperm, the number indicates the days after fertilization. Data are given as mean ±SD. (n=3). OsActin was used as the control.

## Slide 11
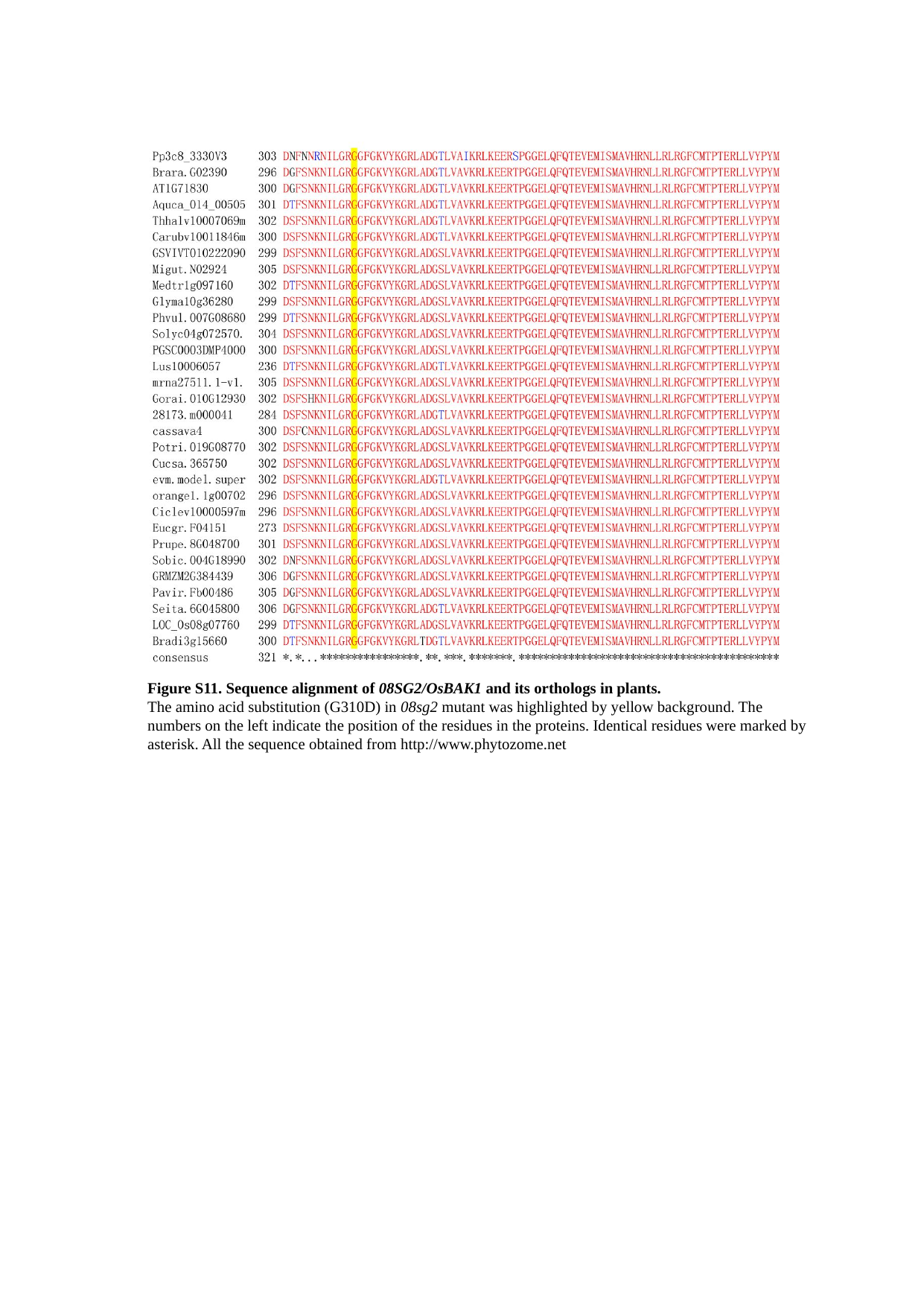

Figure S11. Sequence alignment of 08SG2/OsBAK1 and its orthologs in plants.
The amino acid substitution (G310D) in 08sg2 mutant was highlighted by yellow background. The numbers on the left indicate the position of the residues in the proteins. Identical residues were marked by asterisk. All the sequence obtained from http://www.phytozome.net

## Slide 12
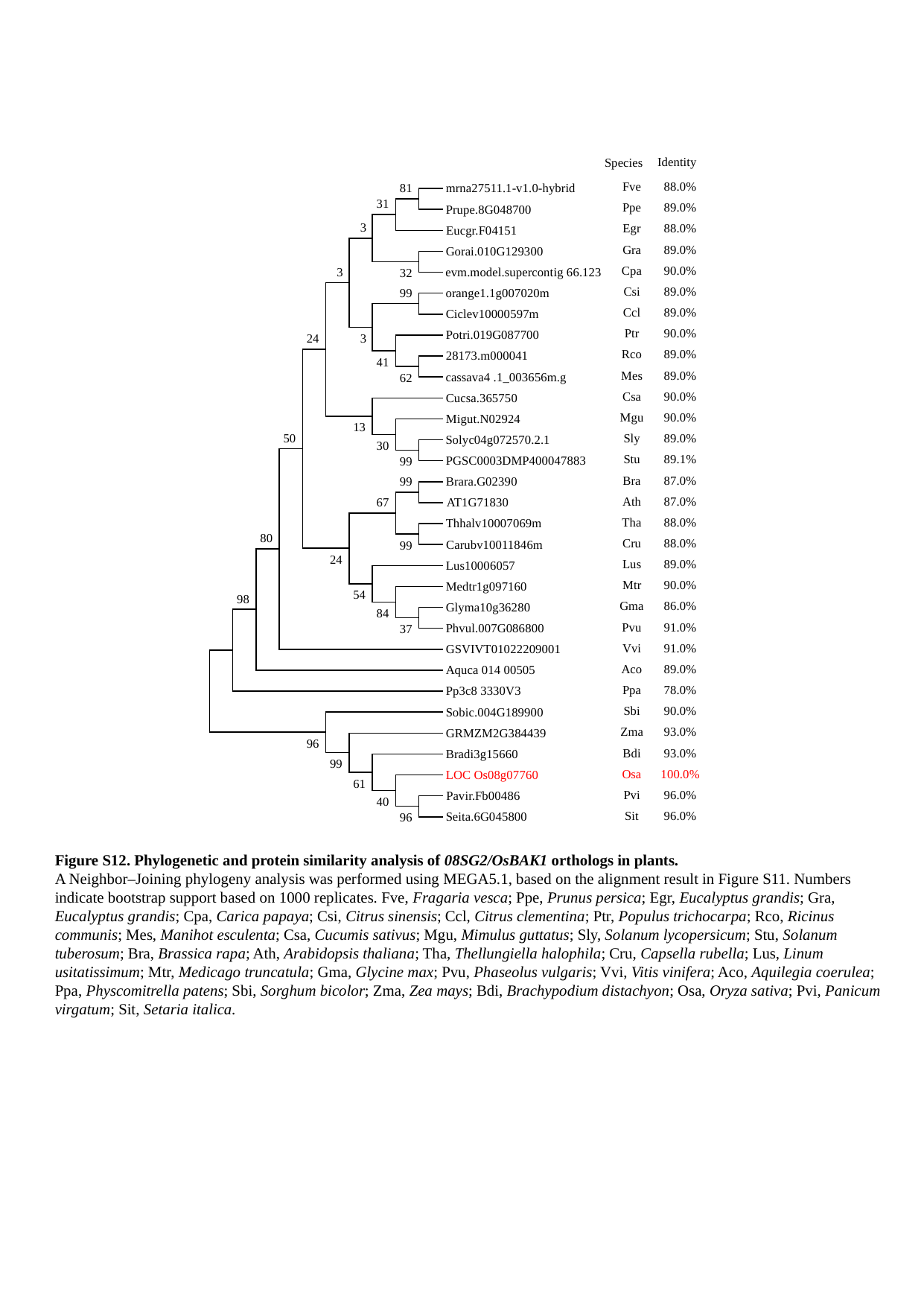

Identity
Species
81
 mrna27511.1-v1.0-hybrid
31
 Prupe.8G048700
3
 Eucgr.F04151
 Gorai.010G129300
3
 evm.model.supercontig 66.123
32
99
 orange1.1g007020m
 Ciclev10000597m
 Potri.019G087700
3
24
 28173.m000041
41
 cassava4 .1_003656m.g
62
 Cucsa.365750
 Migut.N02924
13
50
 Solyc04g072570.2.1
30
 PGSC0003DMP400047883
99
99
 Brara.G02390
67
 AT1G71830
 Thhalv10007069m
80
 Carubv10011846m
99
24
 Lus10006057
 Medtr1g097160
54
98
 Glyma10g36280
84
 Phvul.007G086800
37
 GSVIVT01022209001
 Aquca 014 00505
 Pp3c8 3330V3
 Sobic.004G189900
 GRMZM2G384439
96
 Bradi3g15660
99
 LOC Os08g07760
61
 Pavir.Fb00486
40
 Seita.6G045800
96
| Fve | 88.0% |
| --- | --- |
| Ppe | 89.0% |
| Egr | 88.0% |
| Gra | 89.0% |
| Cpa | 90.0% |
| Csi | 89.0% |
| Ccl | 89.0% |
| Ptr | 90.0% |
| Rco | 89.0% |
| Mes | 89.0% |
| Csa | 90.0% |
| Mgu | 90.0% |
| Sly | 89.0% |
| Stu | 89.1% |
| Bra | 87.0% |
| Ath | 87.0% |
| Tha | 88.0% |
| Cru | 88.0% |
| Lus | 89.0% |
| Mtr | 90.0% |
| Gma | 86.0% |
| Pvu | 91.0% |
| Vvi | 91.0% |
| Aco | 89.0% |
| Ppa | 78.0% |
| Sbi | 90.0% |
| Zma | 93.0% |
| Bdi | 93.0% |
| Osa | 100.0% |
| Pvi | 96.0% |
| Sit | 96.0% |
Figure S12. Phylogenetic and protein similarity analysis of 08SG2/OsBAK1 orthologs in plants.
A Neighbor–Joining phylogeny analysis was performed using MEGA5.1, based on the alignment result in Figure S11. Numbers indicate bootstrap support based on 1000 replicates. Fve, Fragaria vesca; Ppe, Prunus persica; Egr, Eucalyptus grandis; Gra, Eucalyptus grandis; Cpa, Carica papaya; Csi, Citrus sinensis; Ccl, Citrus clementina; Ptr, Populus trichocarpa; Rco, Ricinus communis; Mes, Manihot esculenta; Csa, Cucumis sativus; Mgu, Mimulus guttatus; Sly, Solanum lycopersicum; Stu, Solanum tuberosum; Bra, Brassica rapa; Ath, Arabidopsis thaliana; Tha, Thellungiella halophila; Cru, Capsella rubella; Lus, Linum usitatissimum; Mtr, Medicago truncatula; Gma, Glycine max; Pvu, Phaseolus vulgaris; Vvi, Vitis vinifera; Aco, Aquilegia coerulea; Ppa, Physcomitrella patens; Sbi, Sorghum bicolor; Zma, Zea mays; Bdi, Brachypodium distachyon; Osa, Oryza sativa; Pvi, Panicum virgatum; Sit, Setaria italica.

## Slide 13
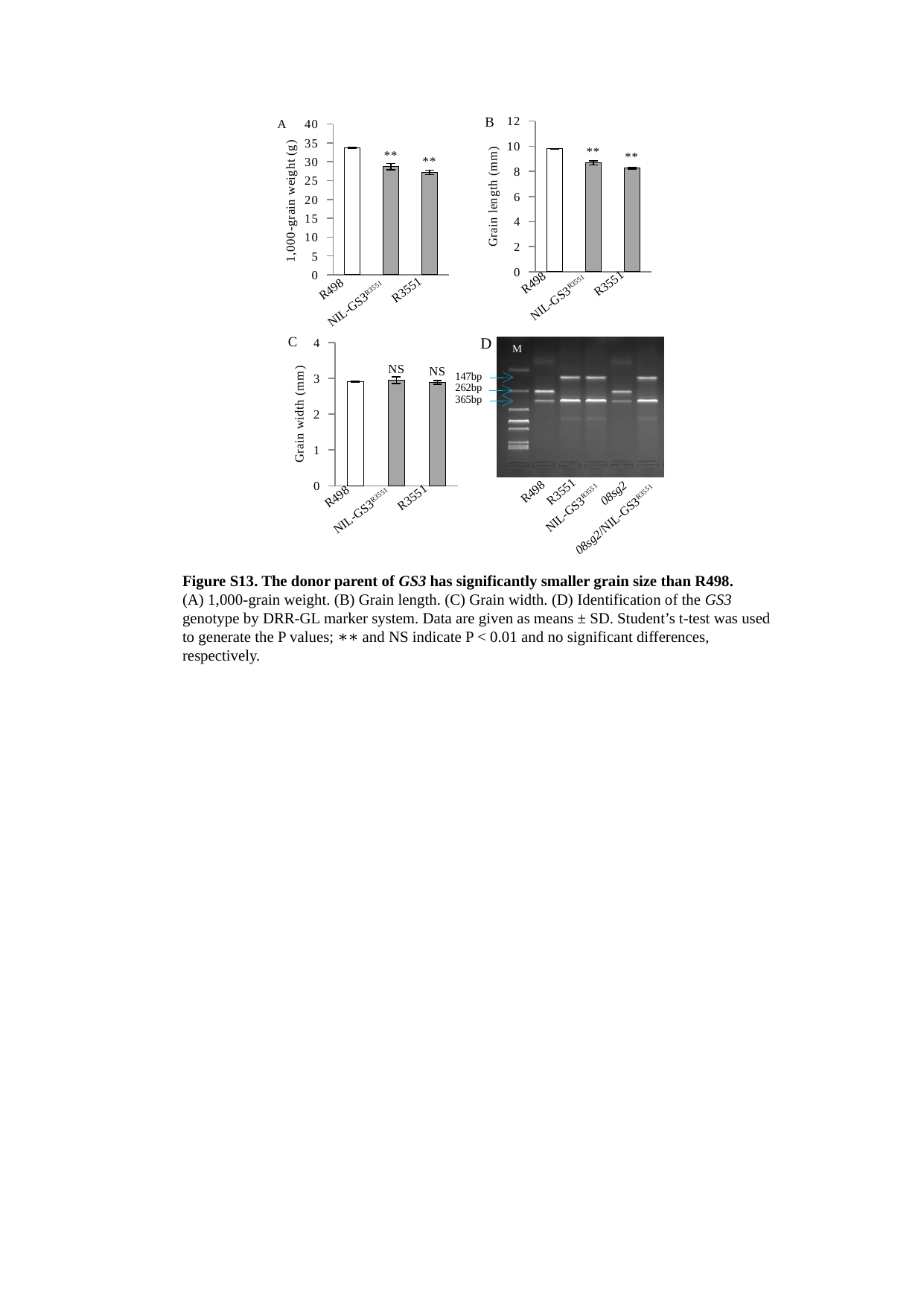

B
### Chart
| Category | |
|---|---|
| R498 | 9.8 |
| NIL-GS3R3551 | 8.67 |
| R3551 | 8.26 |R498
R3551
NIL-GS3R3551
A
### Chart
| Category | |
|---|---|
| R498 | 33.69000000000001 |
| NIL-GS3R3551 | 28.71 |
| R3551 | 27.189999999999987 |R498
R3551
NIL-GS3R3551
C
### Chart
| Category | |
|---|---|
| R498 | 2.9099999999999997 |
| NIL-GS3R3551 | 2.9499999999999997 |
| R3551 | 2.8899999999999997 |R498
R3551
NIL-GS3R3551
D
M
147bp
262bp
365bp
08sg2
R3551
R498
NIL-GS3R3551
08sg2/NIL-GS3R3551
Figure S13. The donor parent of GS3 has significantly smaller grain size than R498.
(A) 1,000-grain weight. (B) Grain length. (C) Grain width. (D) Identification of the GS3 genotype by DRR-GL marker system. Data are given as means ± SD. Student’s t-test was used to generate the P values; ∗∗ and NS indicate P < 0.01 and no significant differences, respectively.

## Slide 14
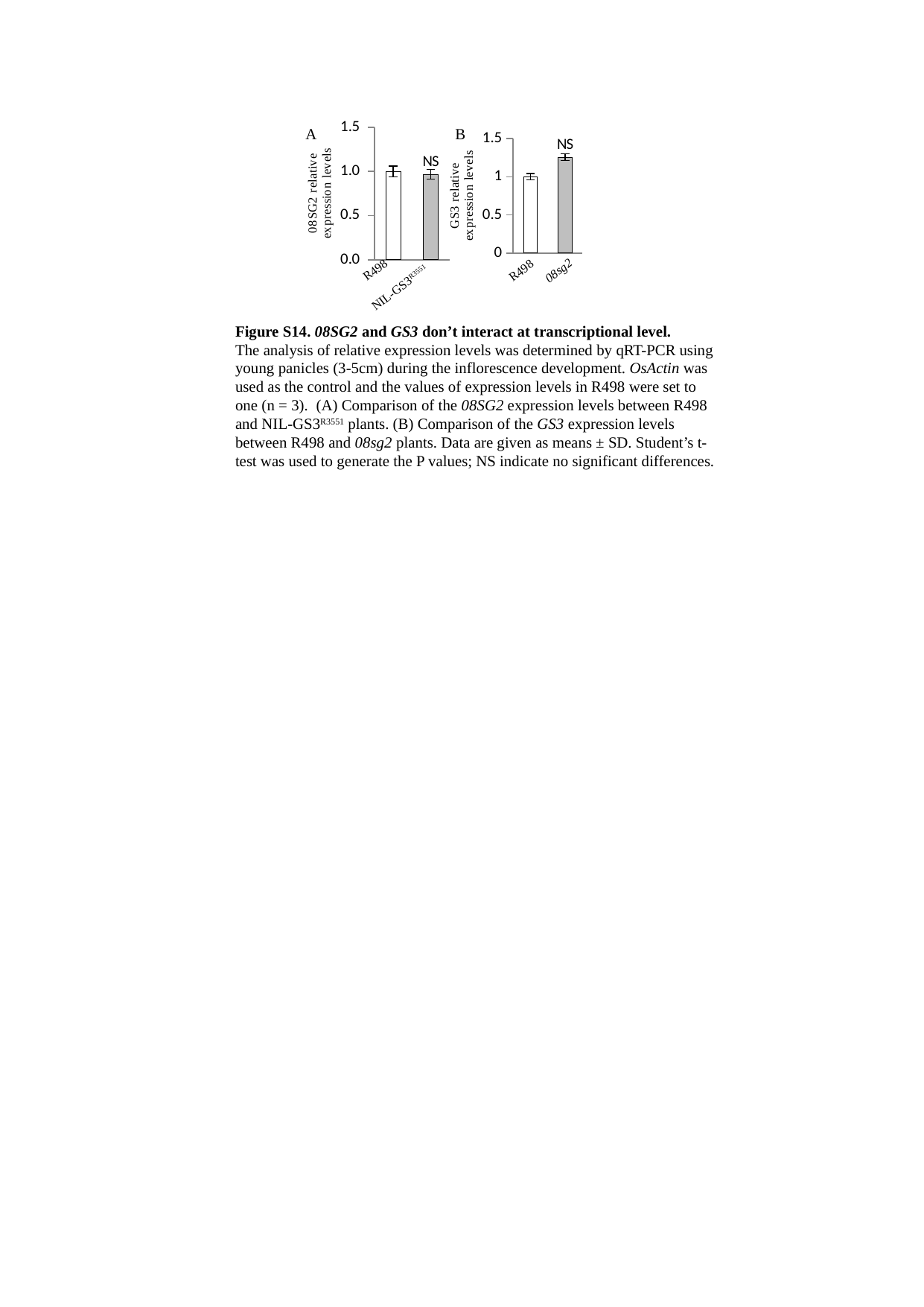

### Chart
| Category | Expression |
|---|---|
| R498 | 1.0 |
| NIL-GS3 | 0.9674445080761 |R498
NIL-GS3R3551
A
B
### Chart
| Category | Expression |
|---|---|
| R498 | 1.0 |
| 08sg2 | 1.25547716164419 |R498
08sg2
Figure S14. 08SG2 and GS3 don’t interact at transcriptional level.
The analysis of relative expression levels was determined by qRT-PCR using young panicles (3-5cm) during the inflorescence development. OsActin was used as the control and the values of expression levels in R498 were set to one (n = 3). (A) Comparison of the 08SG2 expression levels between R498 and NIL-GS3R3551 plants. (B) Comparison of the GS3 expression levels between R498 and 08sg2 plants. Data are given as means ± SD. Student’s t-test was used to generate the P values; NS indicate no significant differences.

## Slide 15
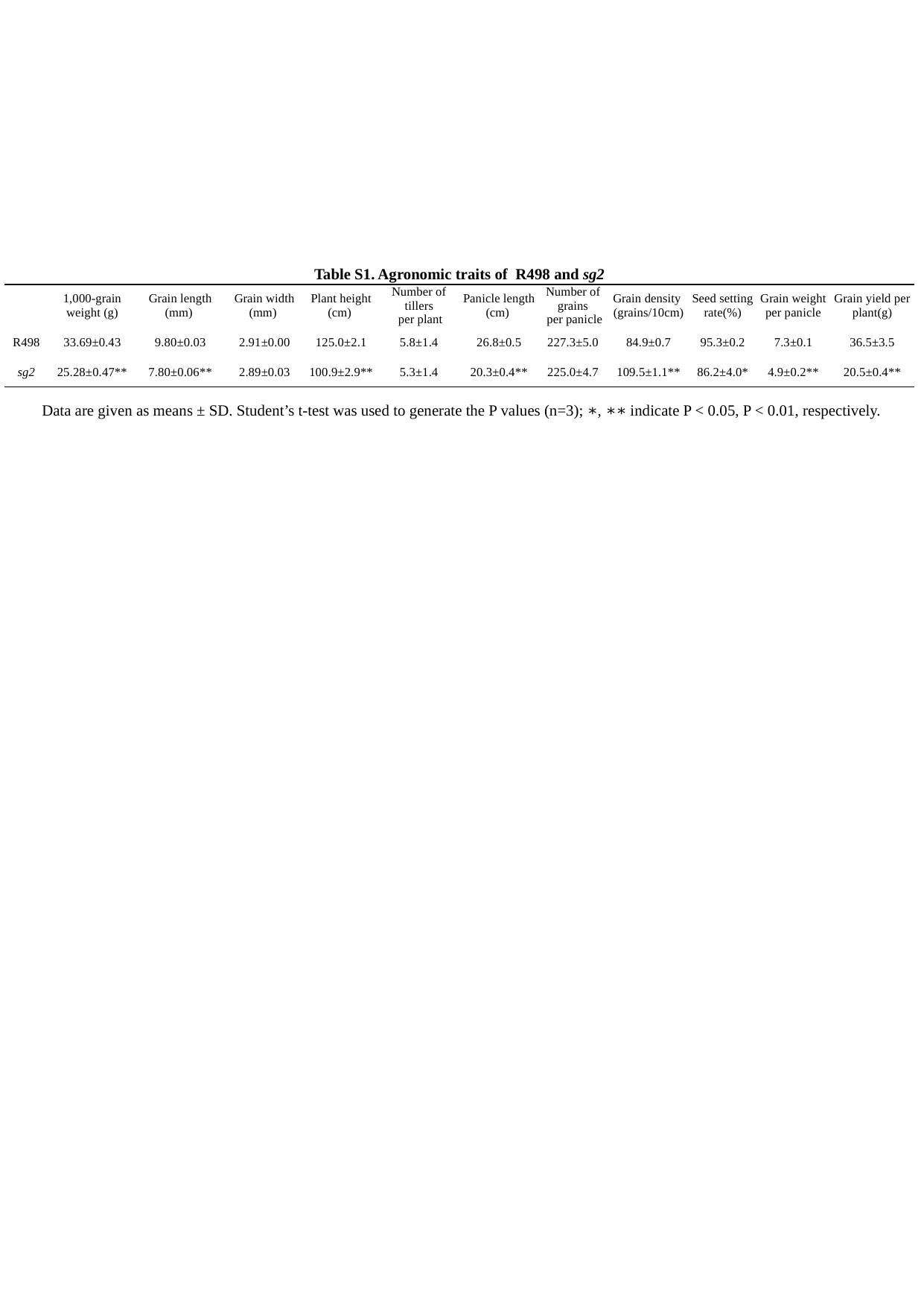

| Table S1. Agronomic traits of R498 and sg2 | | | | | | | | | | | |
| --- | --- | --- | --- | --- | --- | --- | --- | --- | --- | --- | --- |
| | 1,000-grain weight (g) | Grain length (mm) | Grain width (mm) | Plant height (cm) | Number of tillers per plant | Panicle length (cm) | Number of grains per panicle | Grain density (grains/10cm) | Seed setting rate(%) | Grain weight per panicle | Grain yield per plant(g) |
| R498 | 33.69±0.43 | 9.80±0.03 | 2.91±0.00 | 125.0±2.1 | 5.8±1.4 | 26.8±0.5 | 227.3±5.0 | 84.9±0.7 | 95.3±0.2 | 7.3±0.1 | 36.5±3.5 |
| sg2 | 25.28±0.47\*\* | 7.80±0.06\*\* | 2.89±0.03 | 100.9±2.9\*\* | 5.3±1.4 | 20.3±0.4\*\* | 225.0±4.7 | 109.5±1.1\*\* | 86.2±4.0\* | 4.9±0.2\*\* | 20.5±0.4\*\* |
Data are given as means ± SD. Student’s t-test was used to generate the P values (n=3); ∗, ∗∗ indicate P < 0.05, P < 0.01, respectively.

## Slide 16
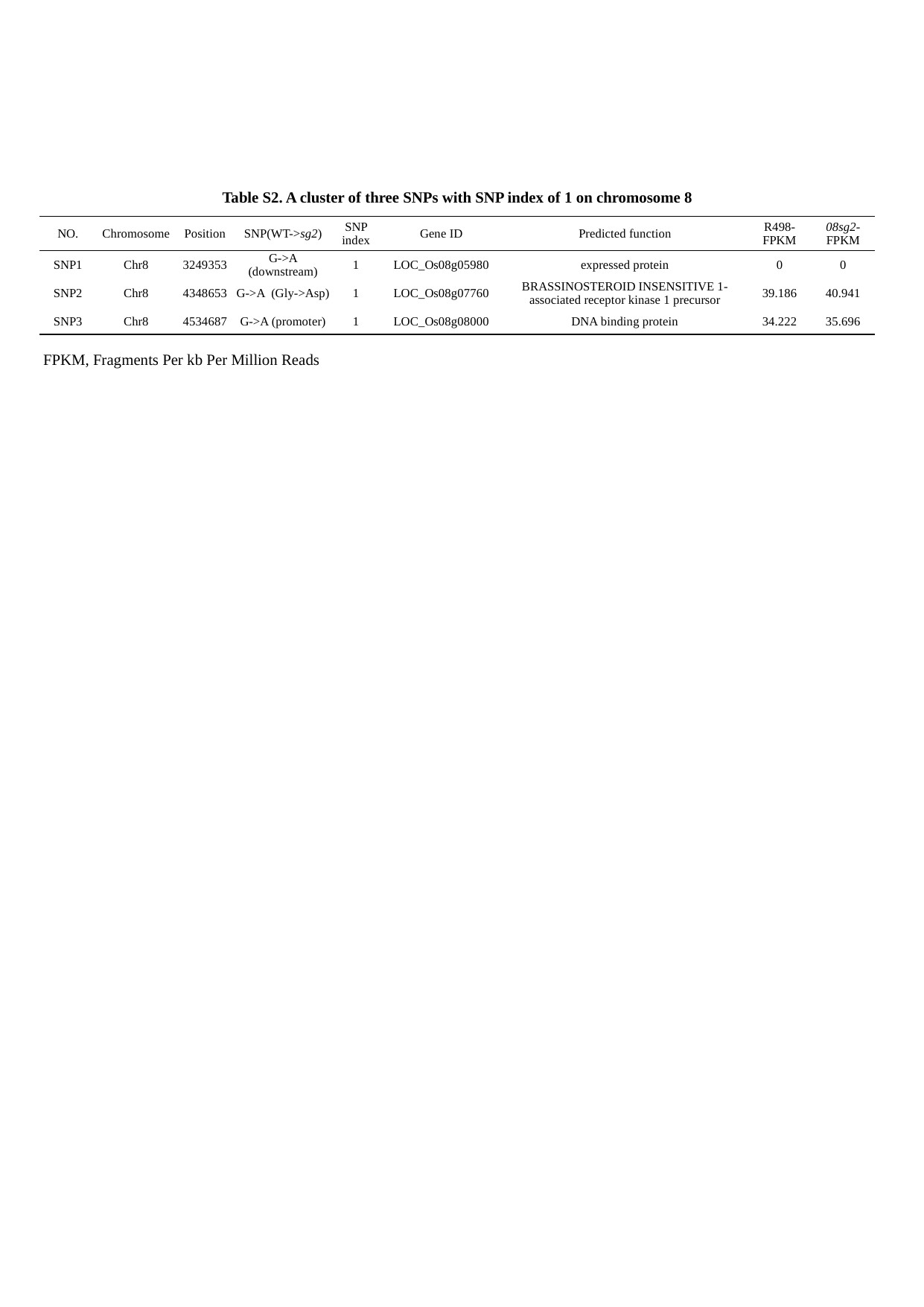

| Table S2. A cluster of three SNPs with SNP index of 1 on chromosome 8 | | | | | | | | |
| --- | --- | --- | --- | --- | --- | --- | --- | --- |
| NO. | Chromosome | Position | SNP(WT->sg2) | SNP index | Gene ID | Predicted function | R498-FPKM | 08sg2-FPKM |
| SNP1 | Chr8 | 3249353 | G->A (downstream) | 1 | LOC\_Os08g05980 | expressed protein | 0 | 0 |
| SNP2 | Chr8 | 4348653 | G->A (Gly->Asp) | 1 | LOC\_Os08g07760 | BRASSINOSTEROID INSENSITIVE 1-associated receptor kinase 1 precursor | 39.186 | 40.941 |
| SNP3 | Chr8 | 4534687 | G->A (promoter) | 1 | LOC\_Os08g08000 | DNA binding protein | 34.222 | 35.696 |
FPKM, Fragments Per kb Per Million Reads
